# Effect of occupant and restraint variability in reclined positions on submarining probability in frontal car crash scenarios

**DOI:** 10.3389/fbioe.2025.1570572

**Published:** 2025-07-09

**Authors:** Erik Brynskog, Jonas Östh, Karl-Johan Larsson, Johan Iraeus

**Affiliations:** ^1^ Department of Mechanics and Maritime Sciences, Chalmers University of Technology, Gothenburg, Sweden; ^2^ Volvo Cars, Gothenburg, Sweden; ^3^ Autoliv Research, Vårgårda, Sweden

**Keywords:** human body model, pelvis, population variance, reclined, submarining, vehicle safety

## Abstract

**Introduction:**

In future autonomous vehicles, a greater seat back recline angle has been suggested to accommodate a more relaxed occupant position. Due to the reclined position, the pelvis rotates rearward resulting in less favorable in-crash pelvis to lap belt interaction. In a crash, this issue can increase the likelihood of the lap belt disengaging from the pelvis and instead loading the abdomen, *i.e.*, submarining. Hence, to enable assessment of submarining prevention measures for reclined occupants in frontal car crash scenarios, it is motivated to enhance the understanding of pelvis to lap belt interaction.

**Method:**

In this simulation study, the submarining outcome of a population of reclined 50%ile (in terms of height and weight) male occupants, subjected to restraint variability in a semi-rigid seat setup, was analyzed through finite element human body model (FE-HBM) simulations (n = 369). To account for the substantial individual variability associated with pelvic shape, a statistical shape model was utilized to predict a large set of random 50%ile male pelvises. Based on select measurements, a sub-sample was drawn from this set (n = 78) for inclusion in the analysis. The simulated submarining outcome and corresponding occupant/restraint parameters were used to generate a metamodel predicting probability of submarining.

**Results:**

The results showed that random variations of a 50%ile male can be comparable with restraint design variability on submarining outcome for reclined occupants. Significant predictors included three from the occupant (pelvis angle, iliac spine hook angle, and H-Point forward/rearward position), and three from the restraint (buckle angle, seat friction, and seat pan angle). Non-significant predictors included occupant soft tissue thickness and fat stiffness, shoulder belt load limit force, and inclusion/exclusion of single lap belt pre-tensioning.

**Discussion:**

In conclusion, this study implies that future vehicle safety ratings, using different versions of a 50%ile male FE-HBM, may be subject to variation in submarining outcome if harmonization of the target occupant anatomy/posture is not established. In addition, based on the response in a semi-rigid seat setup, this study also indicates that to achieve robust submarining protection for reclined occupants, the current legal requirements on buckle angle might need a shift towards more vertical angles.

## 1 Introduction

Advanced Driver Assistant Systems (ADAS) and Autonomous Vehicles (AV) are expected to reduce the risk of future vehicle collisions (Östling et al., 2019). However, in the foreseeable future, they cannot be expected to avoid all crash scenarios. Simultaneously, the introduction of AV is expected to produce new occupant postures and seat configurations (Jorlöv et al., 2017; Östling et al., 2017), which will introduce a host of challenges for occupant restraint systems. One such change is the ability to recline the seatback to a relaxed posture and move the seat away from the instrument panel. Due to the reclined position, the pelvis rotates rearward resulting in less favorable in-crash pelvis to lap belt interaction (Reed and Ebert, 2018), which can increase the likelihood of the lap belt disengaging from the pelvis as the occupant moves forward and under the lap belt in a frontal car crash scenario resulting in abdominal loading, *i.e.*, submarining. Also, an excessive distance between the occupant and the front structures effectively removes the ability to introduce alternative load paths via airbags or bolsters when constraining the occupant (Rawska et al., 2019). As a result, the reliance on pelvis to lap belt interaction will increase when designing future occupant restraint systems, while starting from a less favorable initial position. Hence, it is motivated to enhance the understanding of this interaction by studying the reclined posture in frontal car crash scenarios.

Retrospective studies on submarining for reclined occupants are not readily available given the limited number of occupants recorded in reclined positions including a known submarining outcome in real-world crashes. Traveling in a reclined position has been associated with increased mortality (Dissanaike et al., 2008) and injury risk (McMurry et al., 2018; Schaefer et al., 2021). However, the small sample size of these studies does not allow for conclusions to be made regarding which extrinsic design parameters might have the greatest potential to mitigate a submarining outcome, or if intrinsic population variability is influential enough to result in different outcomes for varying cohorts of the vehicle occupant population. An alternative approach is to use post-mortem human subject (PMHS) experiments. Several such experiments have recently been conducted (Baudrit et al., 2022; Guettler et al., 2023; Lopez-Valdes et al., 2024; Richardson et al., 2020; 2023; Shin et al., 2023; Somasundaram et al., 2022; 2023; UMTRI AVOK-Series, 2020) which are suitable for validation of physical and numerical surrogate models. However, these experiments are complicated and expensive and, hence, restricted to only exploring part of the possible parameter space when considering both population and restraint variability. To effectively explore submarining for reclined occupants in a more complete representation of the available parameter space, prospective models utilizing computer simulations represent a good alternative.

A common prospective tool used in current vehicle safety assessments are the Anthropometric Test Devices (ATDs), a mechanical system designed to replicate human responses in vehicle crash scenarios. However, current ATDs have been shown to lack the function to reliably predict submarining outcomes (Guettler et al., 2023). Alternatively, finite element human body models (FE-HBMs) can be used. FE-HBMs are models designed to replicate actual anatomy and material properties of the human body and are equipped to predict human kinematics, kinetics, and internal strains from omnidirectional loads, as recognized by consumer crash safety rating organizations, such as European New Car Assessment Programme (Euro NCAP). Once viable HBMs become available, Euro NCAP has stated that they will complement their crash testing with FE-HBM evaluations (Van Ratingen and Jacobsen, 2022). Contemporary examples of FE-HBMs include the Total Human Model for Safety (THUMS) (Matsuda et al., 2023), the Global Human Body Model Consortium (GHBMC) (Gayzik et al., 2012), the VIVA+ (John et al., 2022), and the SAFER HBM (Pipkorn et al., 2021). Of these, the SAFER HBM is most suitable for this study since it comprises a comprehensive published evaluation of model responses in a variety of submarining scenarios (Brynskog et al., 2024), and has the capability of representing human pelvic bone shape variability based on a statistical shape model for its pelvis mesh (Brynskog et al., 2021; 2022).

All FE-HBMs presented above are defined for specific cohorts of the population, *e.g.*, 50%ile male/female, 5%ile female, 95%ile male, however, they are based on varying assumptions regarding their underlying anatomical reference. For instance, the GHBMC and THUMS models have utilized the geometry of specific subjects, defined by a selection of metrics to represent each population cohort, to build their FE-HBMs. While this method has the benefit of representing the anatomy of a complete human being, a disadvantage is that each cohort of the population is only represented by a sample of one. With regard to the pelvis specifically, it has been shown that interpersonal variations can be even greater than the average difference between sexes, and by predicting pelvic shape based on sex, age, stature and body mass index (BMI), only 29% of the total pelvic shape variance is captured (Brynskog et al., 2021). Hence, although the subjects used as reference can be considered representative of their population cohort by certain metrics, such as height and weight, guarantee that they represent the cohort’s average pelvic shape, may not be granted. A different approach was applied when developing the SAFER HBM, in which the average shape of anatomical features, *e.g.*, rib cage and pelvic bone, were computed from a population of geometrical samples (Brynskog et al., 2022; Iraeus and Pipkorn, 2019). FE models of each anatomical feature are then built utilizing its average geometry, while population variability is considered through FE mesh morphing (Brynskog et al., 2022). However, a drawback with this method is that potential correlations between individual interacting features could be lost.

The reasons for differences in pelvis to lap belt interaction for reclined occupants vary considerably. Besides inherent differences in pelvic bone structures, stemming from subject anthropometry, material diversity such as fat and muscle stiffness (Naseri et al., 2018) contributes to interaction differences, as does differences due to subject preference, such as posture and belt placement (Reed and Ebert, 2018), as well as restraint differences, *e.g.*, seat stiffness, seat angle, and belt anchor-point position. Previous studies have investigated the effect of varying global anthropometry (5%ile female/50%ile male/95%ile male), posture, material properties, pelvic shape, and vehicle environment (Boyle et al., 2019; Brynskog et al., 2024; Grébonval et al., 2021; Rawska et al., 2019; 2020; 2021; Rees et al., 2023; Richardson et al., 2024; Takeuchi et al., 2024). Only one study (Rees et al., 2023) included population based pelvic shape variations, however, they were limited to local variations at the anterior superior iliac spine (ASIS) and preliminary results have to date only been presented in a short communication. Including work done for a normal upright seated posture, some additional sources can be added which include differences in pelvic shape or the soft tissue around the hips (Izumiyama et al., 2020; Mizuno et al., 2019; Naseri et al., 2022). However, the variations were either investigated one-at-a-time in a single vehicle environment or in a simplified static setup that warrants additional analysis in a full-scale dynamic setup. Summarizing the conclusions of the above references, higher submarining risk appears to be associated with small females, greater pelvis angle, smaller pelvises, less belt-to-pelvis overlap, more horizontal seats, more horizontal lap belts, and less restriction on the pelvis forward motion through alternative load paths, *e.g.*, knee or foot supports. Simultaneously, conflicting results have been identified regarding the effect of BMI as certain studies have found that higher BMI leads to increased submarining risk (Naseri et al., 2022; Uriot et al., 2006), explained by greater shearing in thicker soft tissues and that the belt is less likely to “hook” the pelvis, while other studies have found a decreased submarining risk (Izumiyama et al., 2020; Mizuno et al., 2019; Richardson et al., 2023), explained by “anchoring” of the belt to the pelvis by the protruding abdomen. If the difference in outcome relates to a variation in belt placement, a static *versus* dynamic setup, inclusion/exclusion of local abdominal geometry, or modeling of the fat material properties remains to be explored.

Although previous research referenced has been valuable for highlighting the challenges in protecting reclined occupants and identifying factors linked to submarining, it does not easily allow comparison of the relative importance of these factors, particularly regarding differences in pelvic shape. Furthermore, it does not explore if variations associated with a 50%ile male are comparable with the expected restraint variability on submarining outcome. This issue could have implications when using different 50%ile FE-HBMs as evaluation tools for new safety systems, in both vehicle design and virtual vehicle consumer ratings.

The aim of this study was to analyze the influence of both occupant and restraint variability on submarining outcome of reclined occupants in a frontal car crash scenario, using a simplified vehicle setup with a semi-rigid seat. Specifically, the study intends to answer if intrinsic occupant variance associated with a predicted 50%ile male are comparable with the external restraint design variance on submarining outcome for reclined occupants. Identified factors which significantly affect the outcome will be presented together with their relative importance and estimated non-submarining zone.

## 2 Materials and methods

The material and methods section were carried out in three steps. Each step is briefly explained below, while detailed explanations can be found in the corresponding method subsections. All simulations were performed using the explicit FE solver LS-DYNA MPP R12.2 (ANSYS Livermore Software Technology, California, United States) on a cluster running either 32 (validation) or 128 (sensitivity study) cores. Scripting and statistical analysis was performed with MATLAB R2021b (Mathworks, Natick, MA, United States), while pre/post processing of FE-simulations were performed with ANSA v24.1.1/META v22.1.5 (Beta CAE Systems, Switzerland). The present study was performed with the SAFER HBM v11 (Iraeus et al., 2024). In all simulations, the global coordinate system was defined with positive X in the forward direction and positive Z pointing down.

Step 1: Validation of the SAFER HBM in a frontal car crash scenario on a semi-rigid seat (Uriot et al., 2015) with a belt-in-seat integrated 3-point belt system, at two backrest angles (upright = 25°/reclined = 45°) and two crash severities (32/50 kph), was performed with respect to PMHS data from University of Michigan Transportation Research Institute (UMTRI) (UMTRI AVOK-Series, 2020) in the US.

**FIGURE 1:**
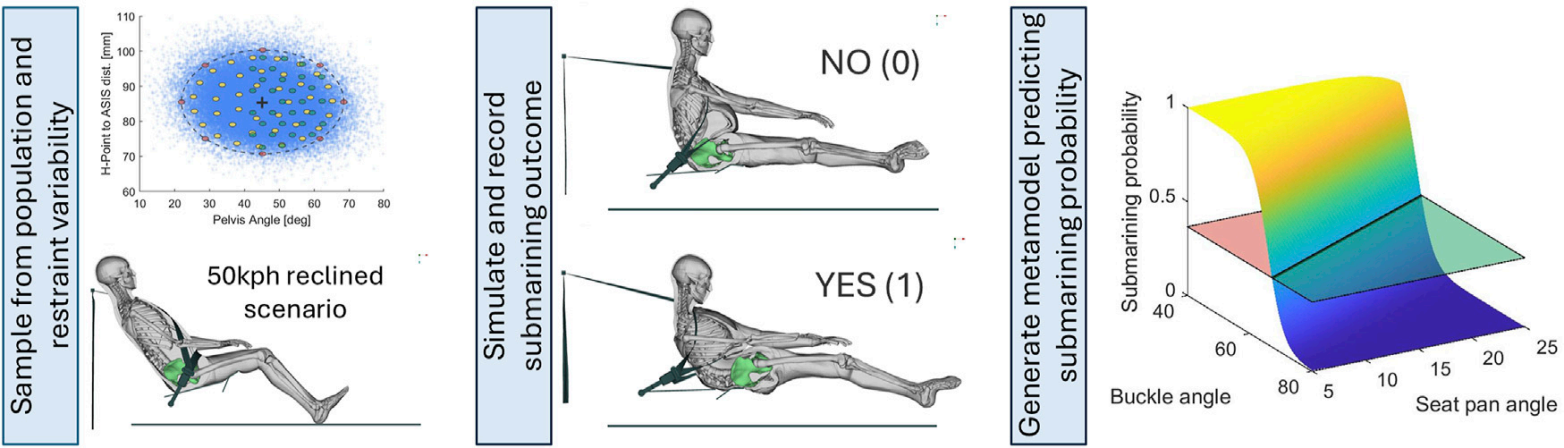
Overview of sensitivity study and metamodel generation. First, sample based on occupant and restraint variability in a reclined frontal car crash scenario. Second, simulation of the complete sample and record submarining outcome (0/1). Third, development of logistic regression metamodel for predicting submarining probability and identifying and comparing significant predictors.

Step 2: A sensitivity study was performed with the validated FE-HBM to generate metamodel training data on submarining outcomes in a reclined frontal car crash scenario. A version of the validation setup was adopted and denoted as baseline, Figure 1, slightly modified to current vehicle restraint settings for the belt while still using the semi-rigid seat. The sampled occupant population was defined based on the residual variance in pelvic shape, fat stiffness, hip soft tissue thickness, and forward/rearward placement on the seat associated with a 50%ile male (age 45 years, stature 1.75 m, weight 77 kg). The restraint variability was based on the expected vehicle design space and current legal requirements. For each sample, a corresponding morphed FE-HBM and a modified FE environment were generated, and the simulated outcome of no submarining/submarining (0/1) at either of the left/right ASIS was recorded.

Step 3: A logistic regression metamodel was generated using the submarining binary outcome as response and the parameter space with main effects as predictors, Figure 1. Convergence of the metamodels training and test errors for increasing sample size were evaluated by 5-fold cross-validation (CV), and significant predictors were identified and compared.

### 2.1 Validation

Previously published PMHS experiment data were downloaded from the National Highway Traffic Safety Administration (NHTSA) database (UMTRI AVOK-Series, 2020). Based on the experiment data and input from UMTRI, a simulation environment was created including sled, distant foot/knee support, semi-rigid seat, and a 3-point belt with buckle, latch, and shoulder belt retractor with pre-tensioner and load limiter functionality, see Supplementary Appendix SA for further details. The validation test series include upright and reclined postures (25°/45° backrest angle) at 32 and 50 kph impact severities. Since the average PMHS (stature 1.73 m, weight 69.3 kg) was smaller than the 50%ile SAFER HBM (stature 1.75 m, weight 77 kg), a scaled version of the SAFER HBM was also evaluated. The scaling was made uniformly in X/Y/Z, using the height ratio, before scaling the density of all soft tissues to match the PMHS weight. The simulation response was qualitatively compared to the reported experiment data, since missing data in the tracked signals did not allow for a quantitative evaluation like the CORrelation and Analysis method (CORA) (Gehre et al., 2009).

### 2.2 Sensitivity study

As baseline, the sensitivity study used a modified version of the 50 kph reclined semi-rigid seat validation setup. The modifications were made to represent a potential future vehicle interior with minimum design constraints from knee support and/or airbags, using current restraint systems and legal requirements. The differences were: no foot/knee supports, sled floor 294 mm below seat H-Point (34 mm higher than in the validation setup), nominal buckle angle to horizontal of 60° (∼85° in the validation), shoulder anchor point position relative to baseline FE-HBM position as defined in (Leledakis et al., 2023) (50 mm rear, 70 mm lateral, and 95 mm higher than the validation), a nominal shoulder belt load limit of 3.5 kN (2.7 kN at 50 kph in the validation), FE-HBM spine and pelvis position targets based on volunteers in reclined postures (Reed and Ebert, 2018) (not the PMHS average from validation). No updates to the semi-rigid seat spring properties were made, as it is currently unknown how future seats will be adopted for reclined occupants and small variations have been shown to have little effect in this setup (Brynskog et al., 2024).

#### 2.2.1 Parameter space

**FIGURE 2:**
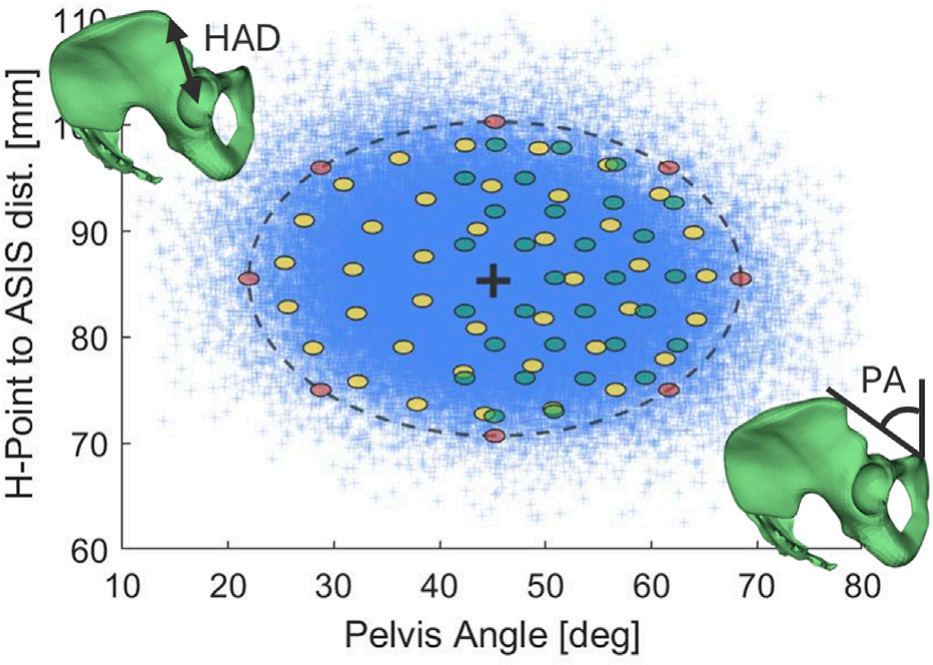
A random sample of 100,000 50%ile male pelvises (blue crosses), with a 95% population boundary for PA and HAD (black dashed line), and the SAFER HBM baseline (black cross). Colored ellipses indicate evaluation regions used to draw a random pelvis when sampling in Batch 1 (red), Batch 2 (yellow), and Batch 3 (green), respectively.

The population was defined to capture 95% of the variability around the 50%ile male prediction by considering pelvic shape, fat stiffness, hip soft tissue thickness, and hip forward/rearward placement (H-Point X-pos.) on the seat. To model the range of pelvic shape variability existing among males considered to be 50%ile in terms of height and weight, a statistical shape model of the pelvis (Brynskog et al., 2021) was used to first generate 100,000 random pelvises. The random sample was achieved by regression residuals in predicted principal component (PC) scores. All generated pelvises were positioned and rotated to align with the sacral endplate and attached with a common spine. Since an unfeasible number of simulations would be required to sufficiently cover the full parameter space of the pelvic shape model (16 parameters), in a subsequent step, the simulated pelvises were selected from the full sample based on how they scored for two resulting measurements. The measurements were pelvis angle (PA), defined as the angle between a line connecting the pubis symphysis (PS) with the ASIS against the vertical axis, and the pelvis H-Point to ASIS distance (HAD), both measured in the mid sagittal plane, see Figure 2. PA was chosen since it has been identified as important for belt/pelvis kinematics and submarining outcome (Izumiyama et al., 2020; Naseri et al., 2022; Takeuchi et al., 2024), while HAD was chosen since it affects the belt-to-pelvis overlap and can be seen as a proxy for pelvis size, which has been identified to influence belt sliding over the ilium and submarining outcome (Izumiyama et al., 2020; Takeuchi et al., 2024). The two measurements were collected from each pelvis and transformed to standard normal form. Using a Chi-squared distribution for two parameters, a 95% population boundary was drawn to be used as sampling limit. For each evaluation point in this space, a sample including ±1° PA and ±0.6 mm HAD (0.1 radius in standard normal form) was collected and a random pelvis with PC scores capped at ±2SD was drawn, see Figure 2 and Supplementary Appendix SB. The fat stiffness variation was defined by three discrete levels (soft/average/stiff) as presented by (Naseri, 2022). The soft tissue thickness around the pelvis was defined as three discrete levels (baseline and ±2RMSE) based on a linear regression model of male buttocks circumference from the ANSUR 2 data (Gordon et al., 2014), see Supplementary Appendix SB. The H-Point X-position on the seat was defined as a continuous variable in the range [−44, +44] mm (±2RMSE) around the seat H-Point, based on regression equation in (Reed and Ebert, 2018).

The restraint variability was defined based on the expected vehicle design space around a reclined occupant and included buckle angle, shoulder belt load limiter (LL), lap belt pre-tensioner (PT), seat friction, and seat pan angle. Buckle angle was defined as a continuous variable, controlled via the left/right anchor-points, to be in the range [40°, 80°] when measured as the angle between a line connecting the belt anchor-points with the seat H-Point against the horizontal (comparable with legal requirements in Regulation No 14 of the Economic Commission for Europe of the United Nations (UN/ECE R14), which restricts the buckle side belt angle to the range [45°, 80°], and Federal Motor Vehicle Safety Standard 210 (FMVSS210), which restricts the buckle side belt angle to the range [30°, 75°]). Shoulder belt LL was defined as a continuous variable in the range [2.0, 5.0] kN (Valdano et al., 2023). Lap belt PT was defined as a categorical variable (OFF/ON), with ON as a 2.0 kN force plateau reached in 1 ms from firing (delayed 8 ms from the shoulder PT, which was kept constant at 1.7 kN peak force triggered 10 ms after acceleration onset with an 8 ms linear ramp) (UMTRI AVOK-Series, 2020; Valdano et al., 2023). Seat friction was defined as a continuous variable in the range [0.2, 0.5] (Brynskog et al., 2024). Seat pan angle was defined as a continuous variable in the range [5°, 25°] (Hagberg and Jodlovsky, 2017), measured against the horizontal axis. See Table 1 for a summary of all parameters.

**TABLE 1:** Summary of all parameters with corresponding ranges.

Parameter	Range [min, base., max]
Occupant	
PA	95% population boundary
HAD	95% population boundary
Fat Stiff	[soft, avg., stiff]
Soft Tissue Thick	[-2RMSE, base., +2RMSE]
H-Point X-pos	[-44, 0, +44] mm
Restraint	
Buckle Angle	[40°, 60°, 80°]
Shoulder Belt LL	[2.0, 3.5, 5.0] kN
Lap Belt PT	[OFF/ON]
Seat Friction	[0.2, 0.35, 0.5]
Seat Pan Angle	[5°, 15°, 25°]

#### 2.2.2 Sampling

The parameter space was sampled in three batches. In short, the purpose of these can be explained as: 1. Evaluating if the design space can produce submarining outcomes by studying parameters at the boundaries, 2. Filling out the design space with samples inside the boundaries, and 3. Adding samples in the space presenting the highest degree of uncertainties following Steps 1 and 2. The three steps were achieved as follows:

1. Only considering the limits and baseline of each parameter (95% population boundary of pelvic shapes and min/max values of other parameters). This step included nine pelvises, Figure 2, and three levels for all other parameters, except the categorical variable lap belt pre-tensioner (ON/OFF), Table 1. Each pelvis was evaluated in the baseline configuration of all other parameters before generating 120 samples using a Latin Hypercube Sample (LHS) algorithm from the MATLAB function “lhsdesign” with a “maximin” criterion. The continuous values of the LHS were rounded to the nearest discrete numeric level of each parameter, *i.e.*, for three levels min = [0, <1/3], baseline = [>1/3, <2/3], max = [>2/3, 1]. In total, this resulted in 129 simulations.
2. To fill out the pelvic shape space, an existing space-filling design of 40 points in a two-dimensional unit disk (Specht, 2024) was utilized. This could be directly applied to the standard normal pelvis measurements by using the radius of the 95% population boundary and resulted in 39 additional random pelvises (the origo was replaced by the baseline), see Figure 2. Fat stiffness, soft tissue thickness, and lap belt pre-tension were kept at their discrete levels defined above, while the remaining variables were treated as continuous. A new sample of 120 points was generated using LHS, but this time the continuous variables were not rounded to any discrete numeric level.
3. A metamodel of the submarining outcome of Batch 1 and 2 was generated and the log-loss function (Equation 3) was evaluated over each parameter range. The parameter range of significant variables was then panned and zoomed to the range with the greatest log-loss values, *i.e.*, presenting the highest degree of uncertainty in the model. As a result, the updated parameter ranges were PA [40°, 63°], H-Point X-position [−18, +35] mm, buckle angle [40°, 64°], seat friction [0.23, 0.47], and seat pan angle [7°, 17°]. The remaining parameter ranges were kept as in Batch 2. The new pelvic shape space was populated by 30 additional points, and a new sample of 120 points was generated using LHS but with a different random seed than for Batch 2.

#### 2.2.3 Model generation

**FIGURE 3:**
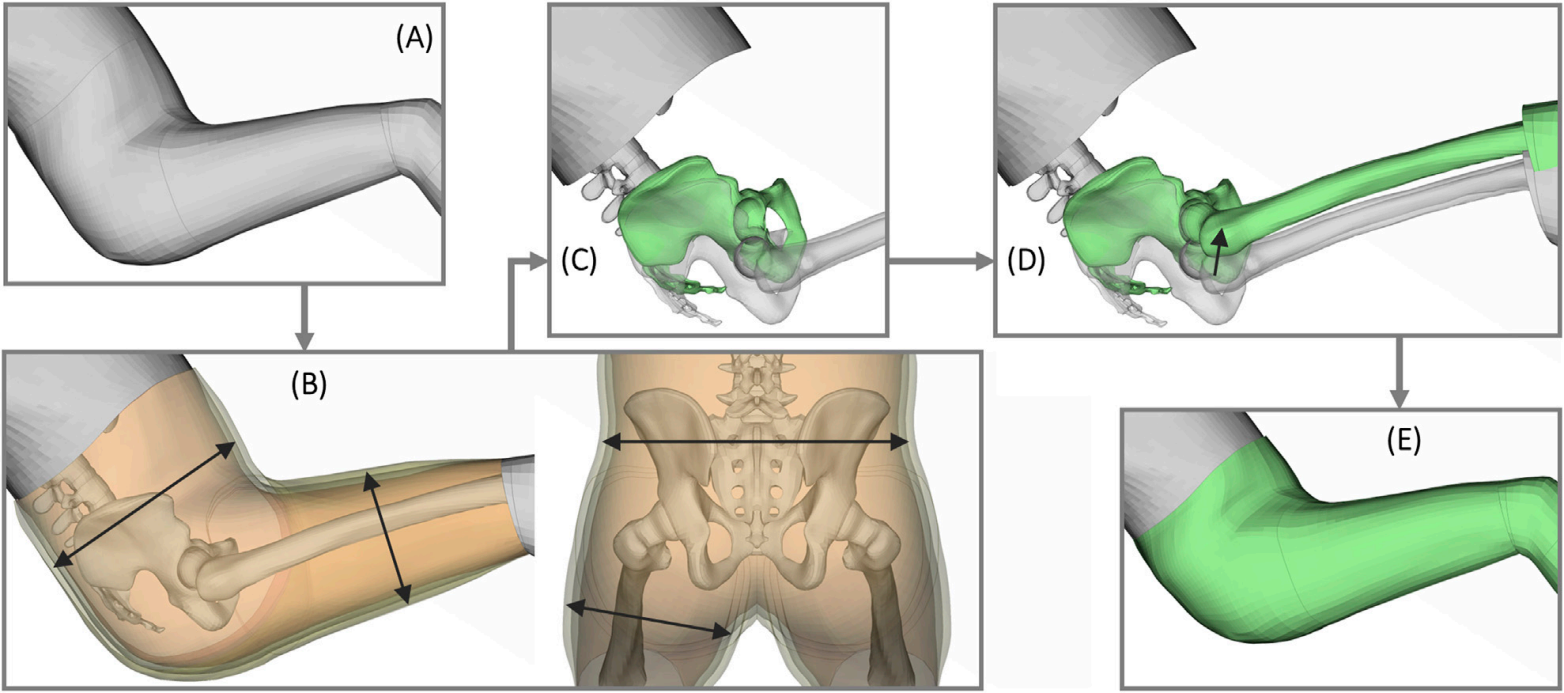
Steps to generate each FE-HBM sample point: **(A)** start from the positioned baseline model, **(B)** create two additional versions of the positioned baseline model with ±2RMSE in buttocks circumference, i.e., three versions of hip soft tissue thickness, **(C)** morph the pelvis to each pelvis geometry sample point in all three soft tissue versions, **(D)** while morphing, move the legs to the predicted hip socket of the morphed pelvis, and **(E)** allow the soft tissue mesh to morph with the new pelvic shape and leg position to generate each FE-HBM sample [in total 234 FE-HBMs (3 soft tissue versions × 78 pelvic shapes)].

To achieve a baseline positioned model, the regression equations from (Reed and Ebert, 2018) was used to predict the posture of a 50%ile male in a seat with a back angle of 45° including a head rest, see Supplementary Appendix SC. The SAFER HBM was then positioned to the computed target using the “Human Body Model–Articulation” tool in ANSA. To generate each FE-HBM sample point, two additional versions of the positioned baseline model were first defined based on varying soft tissue thickness around the hip, see Figure 3 and Supplementary Appendix SB. For the ±2RMSE versions, the skin surface from umbilicus to two-thirds along the thigh were normally projected by −7.7/+8.2 mm using the “DFM” tool in ANSA. Each pelvic shape was then morphed (Brynskog et al., 2022) in all three soft tissue variations of the positioned baseline model. When morphing, the spine was kept fixed and the predicted sacral endplate of each pelvis was aligned with the inferior surface of the S1L5 disc, while the legs were translated to fit in the predicted hip socket position. To have the skin match each pelvis, the outer surface was free to deform with the predicted pelvic shape. The final morphed models were de-penetrated in all contacts and checked for negative volume elements.

#### 2.2.4 Simulation

To initiate each simulation, the FE-HBM was positioned at a target H-Point X-position relative to the seat H-Point (estimated by UMTRI at 95 mm forward and 127 mm above the right rear corner of the seat pan) and moved up in the Z-direction, with the seat belt, until there was no contact between the FE-HBM, seat pan, and/or belt. A gravity load was then applied to reach force quasi equilibrium in 300 ms of simulation time, combined with close to critical global damping. During gravity settling, the belt was re-positioned to the target anchor points and the FE-HBMs head, T1, T8 and shoulders were translated forward/backward to the baseline X-position while constraining the H-Point X-position, to mimic a fixed backrest for all cases while allowing for variations in hip forward/rearward position on the seat. By this approach, the pelvis was free to rotate while settling to reach the equilibrium associated with the current occupant and restraint configuration. In the final 50 ms of gravity settling, the damping was gradually ramped down to zero before applying the actual crash pulse. To evaluate the occurrence of submarining in the crash phase, the position of the midline of the belt in the left/right ASIS plane was evaluated together with the pelvis H-Point X-velocity, as described in (Brynskog et al., 2024). If the belt midline at any time was found above and behind either of the left/right ASIS, while the pelvis still had a forward velocity, the result was flagged as submarining. This definition was shown to give a robust prediction of submarining occurrence for the SAFER HBM when compared to previous PMHS experiments (Brynskog et al., 2024).

### 2.3 Metamodeling

A metamodel describing the submarining binary outcome response (0/1) as a function of sampled input parameters (predictors) can be analyzed as a logistic regression problem where the probability of the outcome, $y_{i}=1$ , is estimated given a known input vector of predictors $x_{i}=\left[x_{1},x_{2},\ldots ,x_{m}\right]$ , where *i* indicates the current sample and *m* the number of predictors. The standard logistic function is defined to output a continuous value, $p\left(x\right)$ , between zero and one, given any real input that is a linear function of one or multiple predictors:

$$p\left(x\right)=\frac{1}{1+e^{-\left(\beta_{0}+\sum_{j=1}^{m}\beta_{j}x_{j}\right)}}$$ (1)

where $p\left(x\right)$ is interpreted as the probability that the response variable $y_{i}=1$ given $x_{i}$ , $\beta_{0}$ is the intercept, and $\beta_{j}$ is the coefficient (or slope) of predictor *j*. The logit (or log odds) is defined as the inverse of the standard logistic function and satisfies:

$$\ln\left(\frac{p\left(x\right)}{1-p\left(x\right)}\right)=\beta_{0}+\sum_{j=1}^{m}\beta_{j}x_{j}$$ (2)

which can be considered as a standard linear regression problem. To find the intercept and coefficient of the linear regression function, *n* pairs of known predictors and responses ( $x_{i},y_{i}$ ) are utilized as training data for the model. This model can then be used to predict probability of the outcome $\hat{p}\left(x\right)$ given new (unseen) input data ${\hat{x}}_{i}$ .

To evaluate the quality of the model, four metrics were considered: Area Under the Curve (AUC), Receiver Operating Characteristic (ROC), accuracy, and log-loss. The AUC score indicates how well the model can distinguish between classes (here 0/1 submarining), with AUC = 0.5 equaling a random guess and AUC = 1.0 a perfect classifier. The threshold for classifying the response was set based on the ROC, and a predicted probability less/greater than the threshold was considered as 0/1 (non-submarining/submarining), respectively. Accuracy was then computed as the ratio of correct predictions, when compared to the true outcome for each simulation, to the total number of cases. The log-loss of the model was defined as:

$$Logloss=-\frac{1}{n}\sum_{i=1}^{n}\left[y_{i}\ln\left(p_{i}\right)+\left(1-y_{i}\right)\ln\left(1-p_{i}\right)\right]$$ (3)

where $y_{i}$ and $p_{i}$ are the true outcome and predicted probability of sample *i*. Essentially, the log-loss evaluates the natural logarithm of the prediction given the true outcome, resulting in zero for a perfect prediction and increasing exponentially the more incorrect the prediction. This can be considered analogous to the Mean Squared Error (MSE) loss function used to evaluate models in standard linear regression.

The convergence of the resulting model for increasing sample size and the ability to predict using a training/test split was evaluated by 5-fold CV. The sample was first split into 20 bins using the MATLAB function “cvpartition,” with a stratified sample based on the submarining response. The sample of the first bin was then split a second time by 5-fold CV (again using “cvpartition” and a stratified sample) to generate training and test data for the current bin. The model was fitted on the training data before the log-loss and accuracy was computed separately on the training and test data, before the process was repeated for all five folds. The second bin of the sample split was then added to the first bin and the process repeated until the entire sample was included. The entire process was repeated 100 times with varying random generation seeds to compute the median, upper, and lower quartiles.

#### 2.3.1 Metamodel parameter selection

Batch 1 was not used when generating the final metamodel since it by design clusters the sample at the limits of each parameter range, making the model less sensitive to the results at the submarining transition and identification of influential parameters biased toward the extremes. Hence, only Batch 2 and 3 were included to fit the logistic regression metamodel using the Matlab function “fitglm”. Furthermore, when first evaluating the metamodel, the sampled PA was found to have less predictive power than the resulting PA after gravity settling, since pelvis rotation from gravity is a result of each occupant and environment interaction. Hence, the sampled PA was replaced as predictor by the resulting PA (correlation score = 0.94), see Supplementary Appendix SB. Also, the sampled HAD was found to have a weak, but opposite than expected effect, on the predicted probability (greater HAD resulted in greater submarining risk). However, a correlation was identified between sampled HAD and iliac spine hook (ISH) angle, defined as the angle between two vectors going from the iliac notch to ASIS/AIIS, respectively (correlation score = 0.71), see Supplementary Appendix SB. Consequently, greater HAD is associated with greater ISH, *i.e.*, a flatter iliac spine. That a flatter iliac spine results in greater submarining risk is in line with the preliminary results by (Rees et al., 2023) and, hence, HAD was replaced by ISH. In summary, predictors considered when generating the metamodel included the parameters described in Table 1, with sampled PA and HAD replaced by resulting PA and measured ISH. All predictors from Table 1 were normalized to be in range [-1, 1] while resulting PA and ISH were transformed to standard normal distribution. The model was generated with significant main effects (p < 0.05) before checking for first order interactions between the significant parameters, with inclusion evaluated by a Bayesian Information Criterion (BIC).

## 3 Results

**FIGURE 4:**
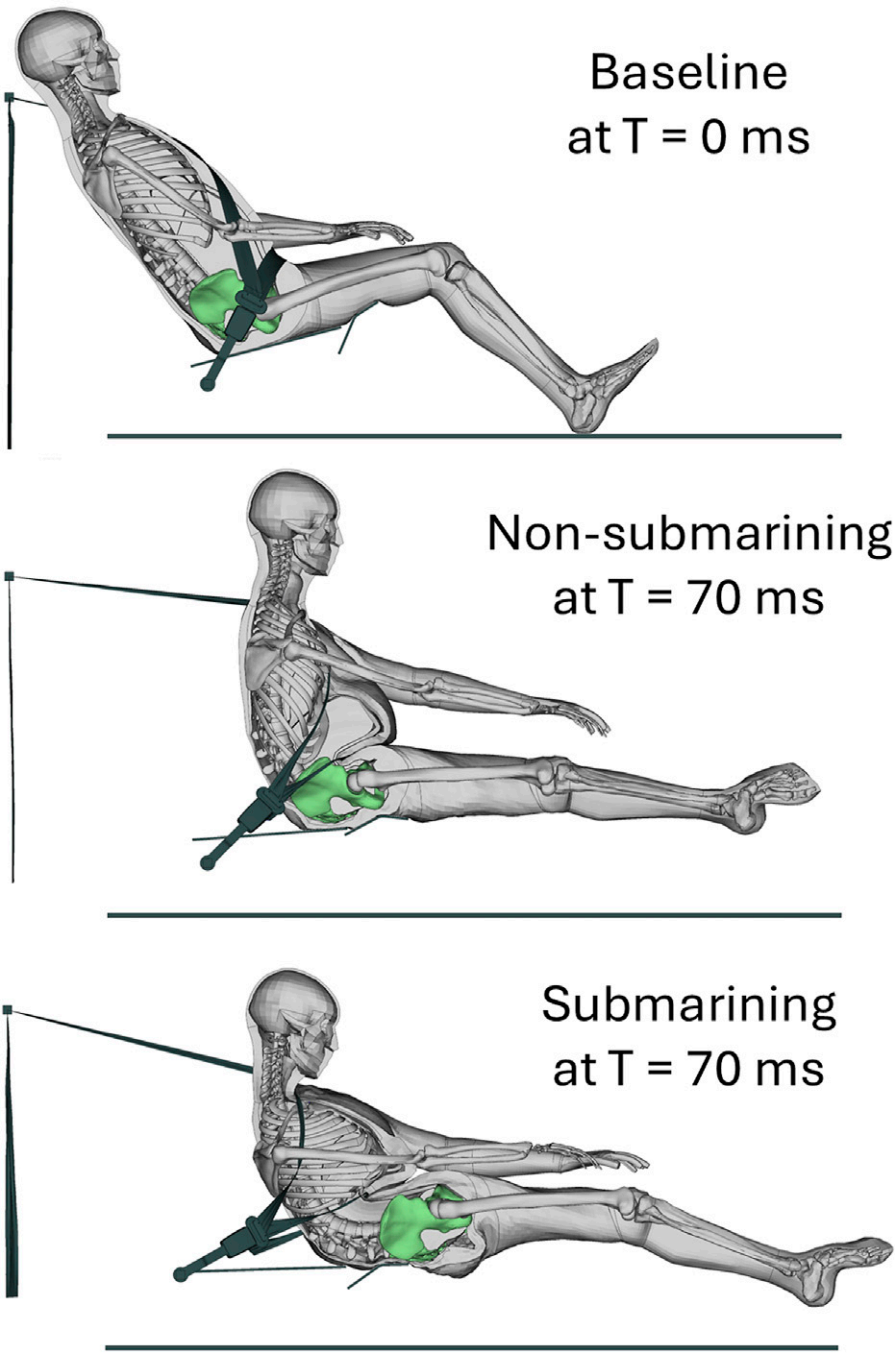
The SAFER HBM in the baseline environment after gravity settling (top), a non-submarining outcome at T = 70 ms (middle), and a submarining outcome at T = 70 ms (bottom).

In total, eight validation simulations and 369 sensitivity simulations were performed in three batches. Of these 369 simulations, 240 were used to build the final metamodel. The 129 simulations from Batch 1 showed that the defined parameter space includes submarining outcomes (28% submarined). Of the simulations used for the final metamodel (Batch 2 and 3, n = 240), 30% resulted in a submarining outcome. All validation runs ended in normal termination while 11% (n = 27) of the simulations used for the metamodel resulted in error termination. However, all error terminations occurred after the submarining classification could be made and were, hence, included in the final metamodel. Figure 4 shows the baseline gravity settled simulation from the sensitivity study together with a non-submarining and submarining response.

### 3.1 Validation

**FIGURE 5:**
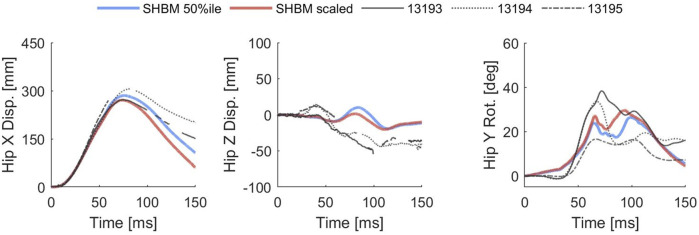
Pelvis kinematic response in the reclined 50 kph validation scenario, PMHS (black), baseline SAFER HBM (blue), and scaled SAFER HBM (red). NOTE: Positive Z points down in the global frame and positive hip Y rotation is rearward, i.e., ASIS rotating back from the lap belt.

Overall, the SAFER HBM reproduced the previously published PMHS responses, with both phase and magnitude for hip X-displacement and hip Y-rotation being mostly in the reported envelope. Hip Z-displacement remained close to zero while the PMHS made a small (≈40 mm) upward movement (positive Z points down in global frame). However, rather than representing an incorrect vertical response for the FE-HBM, most of the difference is explained by the slight over rotation of the seat pan in the model. Figure 5 shows the pelvis kinematics for the reclined 50 kph scenario, while all remaining validation responses can be found in Supplementary Appendix SB. Main deviations from the experiments were identified in the rebound phase, when the submarining outcome was already assessed.

### 3.2 Sensitivity study and metamodeling

**FIGURE 6:**
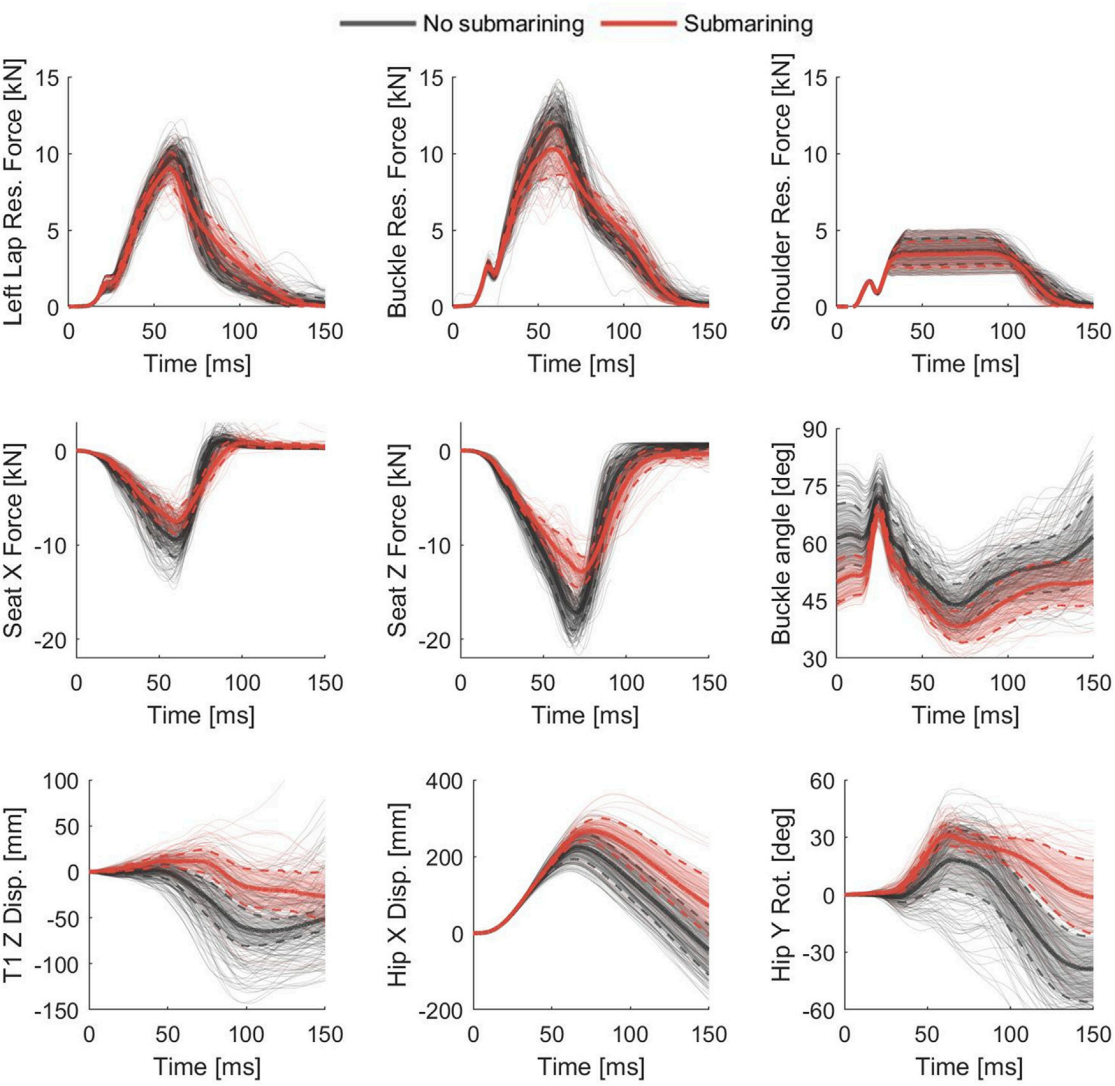
Sample of kinetic and kinematic signals from running Batch 2 and 3 in the sensitivity study. Non-submarining outcomes are colored black while submarining outcomes are colored red, the thick solid/dashed lines are the average/±1SD of the respective groups.

Figure 6 shows a sample of kinetic and kinematic signals from running Batch 2 and 3 in the sensitivity study. Non-submarining outcomes are colored black while submarining outcomes are colored red. The average non-submarining and submarining outcomes together with ±1SD are highlighted by thick lines. The average responses show that submarining outcomes generally have more horizontal initial buckle angles, less force when interacting with the seat, more downward movement of the upper body, more forward movement of the lower body, and more rearward rotation of the pelvis.

**FIGURE 7:**
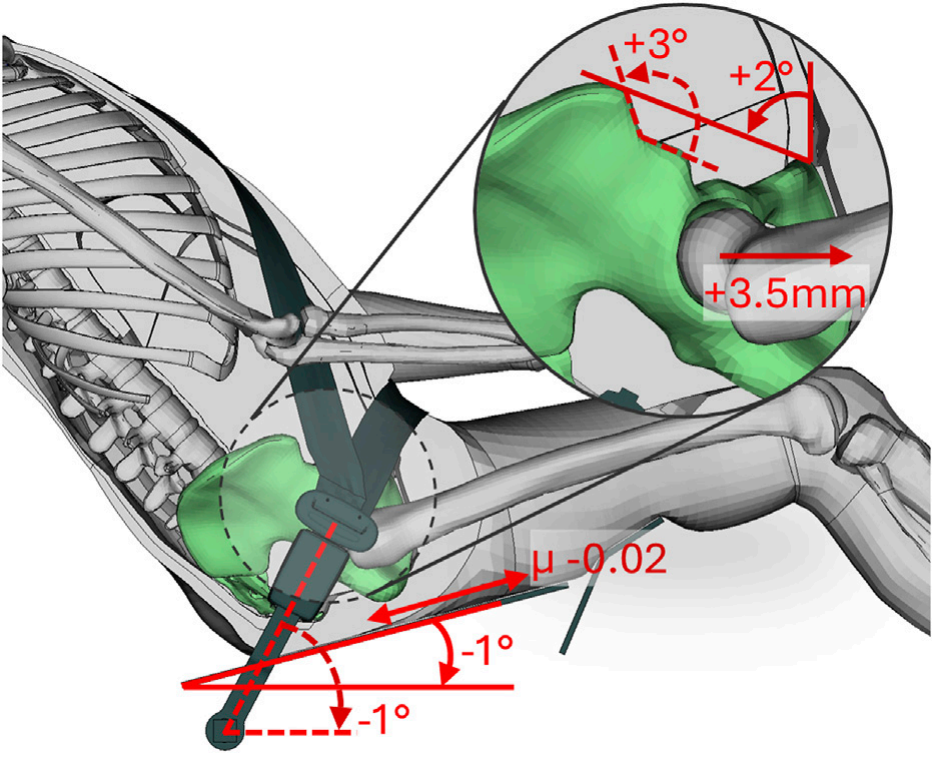
Significant parameters from the metamodel resulting in an equivalent increase in predicted submarining probability when changed one-at-a-time.

Table 2 shows the resulting logistic regression model with corresponding transformation equations for each parameter (used to center and normalize the variance). Resulting PA, ISH, H-Point X-pos., buckle angle, seat friction, and seat pan angle were identified as significant, while fat stiffness, soft tissue thickness, shoulder belt LL, and lap belt PT were identified as non-significant. Generating the model with only the significant parameters and allowing first order interactions added no significant interactions, as evaluated by BIC. Doing one-at-a-time variations of the significant predictors showed that an equivalent increase in predicted submarining probability was achieved by +2° PA, +3° ISH, +3.5 mm H-Point X-pos., −1° buckle angle, −0.02 seat friction, or −1° seat pan angle, see Figure 7.

**TABLE 2:** Logistic regression metamodel for submarining probability prediction with equations for parameter transformation.

Coefficients	Slope	SE	p-val	Parameter Transformation Eq
Intercept	*−6.22*	1.00	*<0.001*	
**PA**	**−2.00**	**0.52**	**<0.001**	$PA_{StdNorm}=\frac{\ln\left(-PA+110\right)-3.6628}{0.2694}$
**ISH**	**−0.65**	**0.31**	**<0.05**	$ISH_{StdNorm}=\frac{\ln\left(-ISH+150\right)-2.4678}{0.4804}$
Fat Stiff	−0.31	0.37	0.39	${FatStiff}_{Norm}=-1\;\left(Soft\right)\;/\;0\;\left(Avg\right)\;/\;1\;\left(Stiff\right)$
Soft Tissue Thick	0.65	0.36	0.07	${SoftThick}_{Norm}=-1\;\left(-2RMSE\right)\;/\;0\;\left(Base\right)\;/\;1\;\left(+2RMSE\right)$
**H-Point X**	**4.94**	**1.00**	**<0.001**	${HPointX}_{Norm}=\frac{HPointX}{44}$
**Buckle Angle**	**−7.95**	**1.26**	**<0.001**	${BuckleAng}_{Norm}=\frac{BuckleAng-60}{20}$
Shoulder Belt LL	−0.34	0.46	0.45	${ShoulderLL}_{Norm}=\frac{ShoulderLL-3.5}{1.5}$
Lap Belt PT	−0.72	0.59	0.22	${LapPT}_{Norm}=0\;\left(OFF\right)\;/\;1\;\left(ON\right)$
**Seat Friction**	**−3.13**	**0.68**	**<0.001**	${SeatFric}_{Norm}=\frac{SeatFric-0.35}{0.15}$
**Seat Pan Angle**	**−4.04**	**0.97**	**<0.001**	${SeatAng}_{Norm}=\frac{SeatAng-15}{10}$

Significant parameters (p < 0.05) are marked in bold text. NOTE: To use the metamodel, each parameter needs to be transformed with the corresponding “Parameter Transformation Eq.” before multiplying by the estimated “Slope”.

**FIGURE 8:**
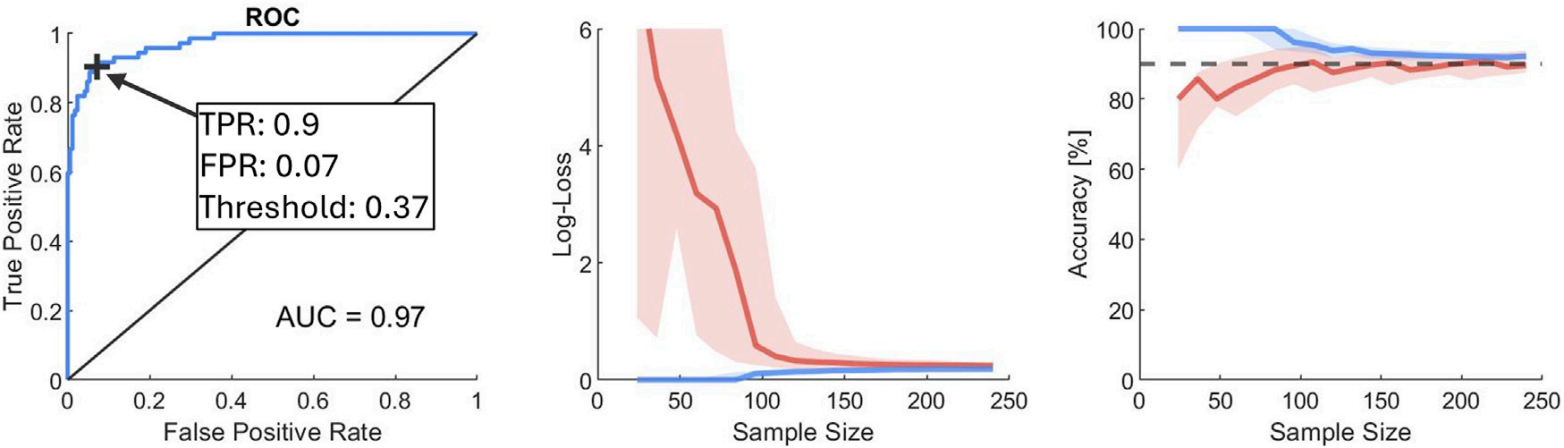
Metamodel ROC/AUC (left), log-loss (middle) and accuracy (right) with increasing sample size. In the ROC plot the threshold for optimal model accuracy is indicated by a black cross. For log-loss and accuracy, red/blue indicates test/training data, respectively, the dashed line represents 90% accuracy, and the envelope represents the lower/upper quartiles.

Figure 8 shows the ROC curve of the metamodel (using the full set of samples) together with convergence for increasing sample size evaluated by log-loss and accuracy on the training and test sets separately. The AUC of the metamodel (using the full set of samples) was 0.97. The optimum threshold for classifying non-submarining/submarining was found at 0.37 with a true positive rate (TPR) of 0.9 and a false positive rate (FPR) of 0.07. The log-loss for the test set converges on an average error in predicted probability of 0.2 (20%), while the model accuracy converges on 90% when using the optimum threshold for classification.

**FIGURE 9:**
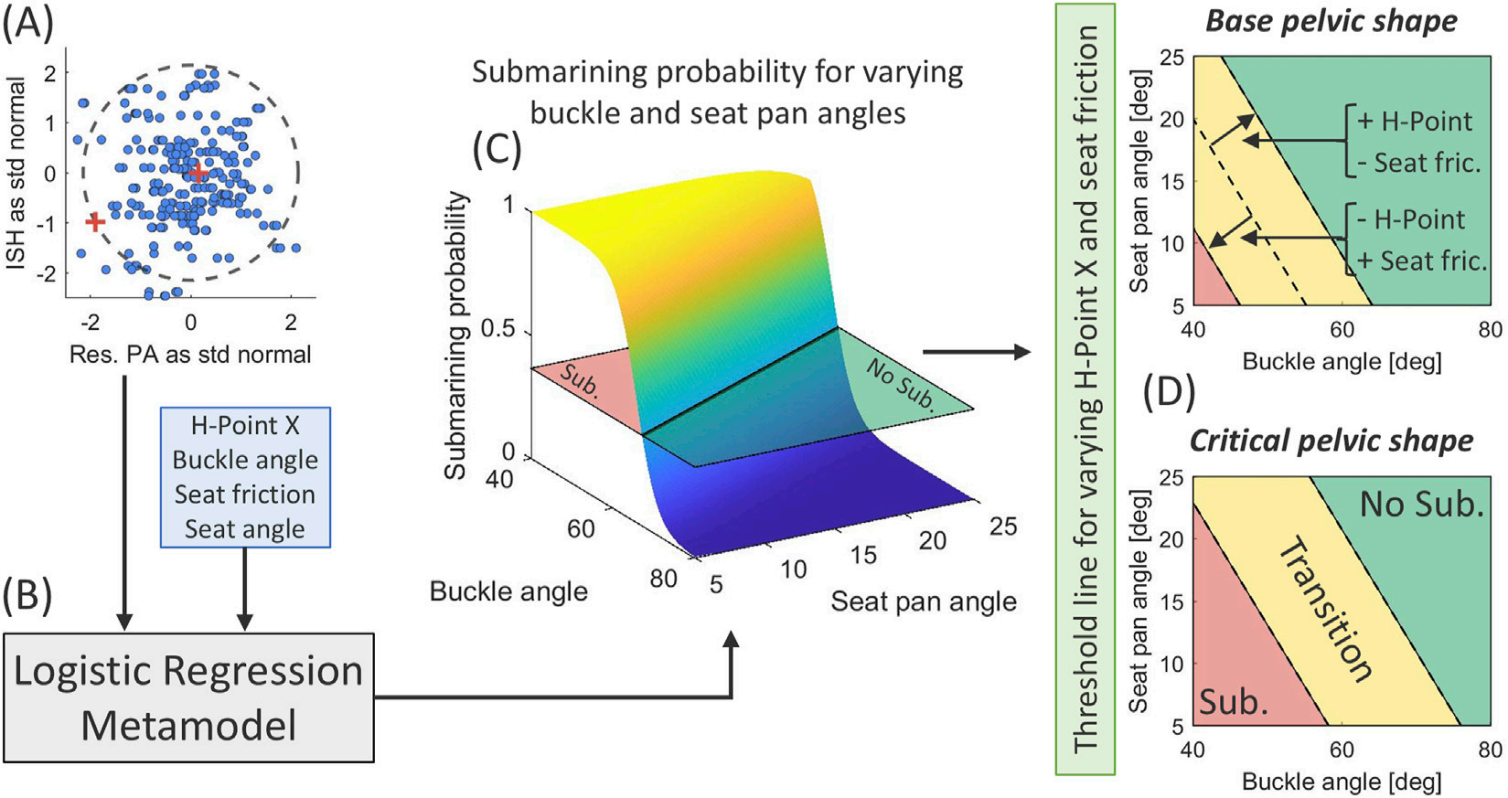
Estimation of the non-submarining/submarining outcome for various buckle and seat pan angles using the baseline and critical pelvic shape. The steps are; **(A)** identifying the baseline and critical pelvic shape (red crosses) by studying the distribution of resulting PA and ISH transformed to standard normal with a 90% population boundary (black dashed curve), **(B)** inputting the pelvic shape and remaining significant predictors into the metamodel, **(C)** plotting the submarining probability response surface for varying buckle and seat pan angles and identifying the threshold line (prob. = 0.37), and **(D)** repeating these steps for varying H-Point X (±22 mm) and seat friction (0.3–0.4) using the baseline and the critical pelvic shapes and identifying non-submarining (green), transition (yellow), and submarining (red) zones.

Figure 9 shows the predicted non-submarining (green)/submarining (red) zone for various buckle and seat pan angle combinations, given the baseline pelvic shape or the critical pelvic shape on the 90% population boundary for resulting PA and ISH. The critical pelvic shape was defined as the point on the boundary that adds the greatest contribution to predicted submarining probability in the metamodel. The transition zone (yellow) indicates a zone where the non-submarining/submarining transition occurs depending on the values of H-Point X-pos. (±22 mm) and seat friction (0.3–0.4). As an example, to predict non-submarining for the critical pelvic shape, seated +22 mm in front of the seat H-Point, with a seat friction of 0.3, and a seat pan angle of 15°, the buckle angle must be greater than 66°. Note that although the continuous response surface is only shown for buckle angle and seat pan angle, the submarining probability can be estimated for all significant parameters using the metamodel presented in Table 2. Showing the effect of all significant parameters simultaneously is not possible, due to the multidimensional continuous parameters space, and can only be done at fixed points/thresholds as in Figure 9D.

## 4 Discussion

In this study, a 50%tile male FE-HBM was validated with respect to reclined occupant responses in frontal car crash scenarios. The FE-HBM was then used to identify if intrinsic occupant variance around a predicted 50%ile male can be comparable with the external restraint design variance on submarining outcome for reclined occupants. To achieve this, 369 frontal car crash simulations on a reclined semi-rigid seat were conducted and the submarining outcome recorded. Based on 240 of these simulations, a metamodel predicting submarining probability was developed to identify significant occupant and restraint predictors. To the best of the authors’ knowledge, this is the first study to comparatively evaluate the effect of both occupant variability, with regard to a predicted cohort of the population, and restraint variability on submarining outcome in reclined frontal car crash scenarios.

### 4.1 Main findings

Occupant variability can be comparable with restraint design variability on submarining outcome for reclined occupants, even when only considering the residual variance associated with a 50%ile male. Consequently, without harmonization of the target occupant anatomy/posture, the results of future vehicle safety ratings using different versions of a 50%ile male FE-HBM could be inconsistent. Of the significant predictors identified, three originated in the occupant geometry and posture (PA, ISH, and H-Point X-pos.), while three originated in the restraint (buckle angle, seat friction, and seat pan angle). The strongest effect on submarining outcome, considering the parameter ranges, originated in the buckle angle, while the remaining parameters had similar contributions, except ISH which was weaker. Using the metamodel, one-at-a-time variations of the significant predictors showed that +2° PA (rearward pelvis rotation), +3° ISH (flatter iliac spine), +3.5 mm H-Point X-pos., relative to the seat H-Point (forward placement), −1° buckle angle (more horizontal), −0.02 seat friction coefficient (less resistance), and −1° seat pan angle (more horizontal), increased the predicted submarining probability equally. A PA standard deviation of 8.7° was measured by (Izumiyama et al., 2018) for male volunteers in an automotive seat (seat back angle 24°). In the present study, using a pelvis statistical shape model (Brynskog et al., 2021) to generate 100,000 random 50%ile male pelvic shapes, a standard deviation in PA of 9.5° was computed. Using these two measurements as reference, and assuming an equal distribution in resulting PA when reclined, having an equal probability of submarining for the average 50%ile male as for a 50%ile male with +2SD in PA, would require compensation of approximately +9° buckle angle, or +5° buckle angle and +4° seat pan angle, or +4° buckle angle and +0.1 seat friction, etc.

Furthermore, the present study found soft tissue thickness and fat stiffness to be non-significant occupant parameters for submarining outcome. As mentioned in the Introduction, previous research is inconclusive regarding the effect of varying soft tissue distribution and properties for submarining prediction. Some have concluded that obesity and a thicker layer of soft tissue increases the risk of the belt sliding over the pelvis (submarining) (Naseri et al., 2022), however, the effect was only visible for BMI >30 kg/m^2^. Hence, this result is consistent with the present study which has evaluated the occupant variability for a 50%ile male with BMI = 25 kg/m^2^. Similarly, sources who have found a decreasing tendency for submarining with higher BMI, have done so for occupants recorded as overweight and obese (BMI >30 kg/m^2^) (Izumiyama et al., 2020; Richardson et al., 2023). Therefore, it is possible that soft tissue properties are non-significant only when smaller variations with regard to BMI = 25 kg/m^2^ are considered, although they may be a stronger contributor for obese occupants. Future work is required to validate obese FE-HBMs in submarining scenarios as well as evaluate the effect of obesity on submarining outcome. Such investigations will require both simulation studies and additional PMHS tests comprising obese occupants.

For the restraint parameters, shoulder belt LL and lap belt PT were found to be non-significant for reclined FE-HBM submarining outcome, when evaluated in the semi-rigid seat environment. This indicates that for a 50%ile male, a shoulder belt LL of 2.0–5.0 kN does not stop the forward pitch of the torso enough to influence submarining outcome. Adding a single lap belt PT, in addition to shoulder belt PT, has been reported to reduce lower extremity and abdominal injury risk in real life crashes, indicating reduced pelvis excursion and submarining risk for upright seated occupants (Couturier et al., 2007; Faure et al., 2007). In the present study, inclusion of a single lap belt PT in addition to the shoulder belt PT showed a trend of reducing submarining risk for the reclined FE-HBMs, although this effect did not reach statistical significance for the simulation sample (p = 0.22). In physical sled testing with an ATD reclined on a production seat, adding a lap belt PT, in addition to shoulder belt PT, did not prevent submarining, while adding a third PT at the buckle side (dual lap belt PT) and moving the belt anchor points forward (corresponding to a more vertical buckle angle) prevented submarining (Östling et al., 2017). Rawska et al. (2020) used a belt with shoulder belt PT and a single lap belt PT and a production seat model in a FE parameter study on different seat back recline angles, seat cushion angles and presence of knee bolster with the 5%ile female and 50%ile male GHBMC HBM models. For upright FE-HBMs, no submarining was predicted regardless of cushion angle and knee bolster, however, for various degrees of reclining, submarining was obtained depending on cushion angle, FE-HBM and presence of knee bolster (Rawska et al., 2020). Thus, the findings regarding the effect of single lap belt PT, buckle angle, and cushion angle are in line with previous results for reclined occupants, evaluated with a production seat environment.

The non-submarining and submarining average (±1SD) kinetic and kinematic responses have been summarized in Figure 6. These responses show that submarining outcome was associated with more horizontal buckle angles, less force when interacting with the seat, greater downward and less forward displacement of the upper torso, and greater forward displacement and rearward rotation of the hip. The association between submarining and a limited seat pan interaction is consistent with the findings by Guettler et al. (2023), who performed PMHS tests in multiple rear seats and found the dominant factors associated with submarining to be related with seat pan geometry.

The results presented in the present study were derived from a model with correct initial lap belt placement, *i.e.,* it was placed below the ASIS in the abdomen-thigh pocket. Compared to belts buckled by a volunteer population (Reed and Ebert, 2018), the simulated lap belt placement is ∼40 mm lower for the top belt edge relative to the right ASIS. However, the placement was approximately in line with the average vertical belt placement found when validating against PMHSs (UMTRI AVOK-Series, 2020). This indicates that the volunteer belt placement may be higher than the professional placement achieved in PMHS experiments. In the present study, submarining FE-HBMs had the belt placed higher relative to the ASIS than the non-submarining FE-HBMs (significant difference in average vertical distance between belt top edge and ASIS at T = 0 ms (p < 0.05, two-tailed t-test)), indicating that real occupants who has secured the belt themselves in a similar scenario could have higher submarining risk than the FE-HBM simulations used to build the metamodel.

**FIGURE 10:**
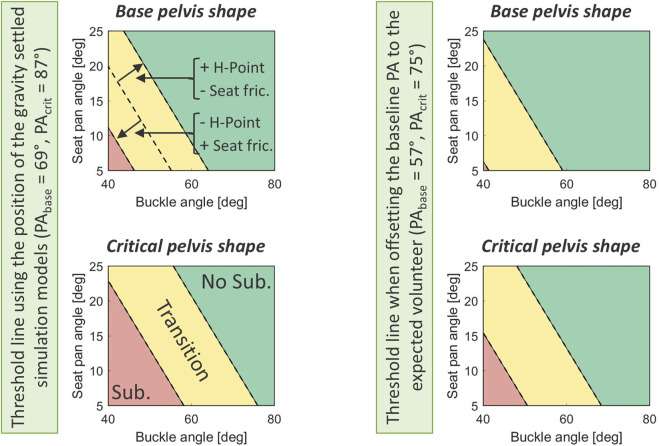
Threshold lines when analyzed in the gravity settled position (left) and when compensated by −12° PA to represent the expected average volunteer from (Reed and Ebert, 2018) (right).

It should also be noted that while the baseline positioned model had a pelvis angle (defined as H-Point to center of S1L5 disc with respect to vertical, PA_HPoint-to-S1L5_) matching the volunteer average of 65° (RMSE = 15.4°) (Reed and Ebert, 2018), the gravity settling caused the pelvis to rotate back by an additional ≈12°, due to the weight of the torso pressing down along the spine, which generated a moment around the pelvis H-point. The pelvis rotation was by design not constrained in the gravity settling phase since the effect of both occupant and restraint variability was part of the aim of this study. Random variations in pelvic shape should, hence, influence the gravity settled pelvis position on a specific seat design. See Supplementary Appendix SC for comparison of the positioned and the gravity settled baseline model. As a result, the average simulated PA_HPoint-to-S1L5_ was 78° (SD = 7.8°, range = [54, 96] °), consequently the analysis overlaps the expected volunteer PA_HPoint-to-S1L5_, emphasizing pelvises with a greater risk of submarining. However, since the simulation range included the expected volunteer position, the metamodel can still be evaluated at this point. Figure 10 shows the effect of evaluating the submarining threshold line for a baseline and a critical pelvic shape using the position of the gravity settled simulation models (as in Figure 9) and with an offset of −12° PA to represent the average volunteer from (Reed and Ebert, 2018). As expected, the threshold line was pushed to the left when decreasing PA, however, a buckle angle greater than 60° was still required to predict a non-submarining outcome for the critical pelvic shape at +22 mm H-Point X-pos., with 0.3 seat friction, and a seat pan angle of 15°, when evaluated on a semi-rigid seat.

### 4.2 Implications for vehicle safety design

The threshold lines and non-submarining zone presented in this study indicates that, to efficiently prevent submarining for a reclined 50%ile male occupant, current legal requirements on buckle angle (45°–80° UN/ECE R14 and 30°–75° FMVSS210) might need a shift towards more vertical angles. These requirements were developed for upright occupants and should, hence, be reconsidered to allow for occupants traveling in a reclined position. Evaluated with a 15° seat pan angle and allowing for some unfavorable variation in pelvic shape, H-Point X-position, and seat friction, the non-submarining zone starts at a buckle angle of around 60°, when evaluated on a semi-rigid seat. With a seat pan angle of 5°, the non-submarining zone starts at a buckle angle greater than 70°, which leaves a very narrow window to the upper legal requirement of 75/80°. Still, these results only account for the residual variance associated with a 50%ile male and preventing submarining for the entire population, including, *e.g.*, the 5%ile female, would be even more challenging given previous research (Rawska et al., 2019; 2020). The scenario evaluated in this study could be considered a possible future vehicle environment providing the manufacturer with the most freedom to design/utilize the space around the occupant, since it does not include any alternative load paths such as knee support, foot support, or airbags. However, restraint systems controlling the lower body forward movement have been shown to reduce submarining tendency (Rawska et al., 2021; Shaw et al., 2018) and could be a key component to effectively prevent submarining for the complete population in reclined scenarios.

The results of this study should not be used as the sole argument for changing current legal requirements on buckle angle without a thorough evaluation of associated consequences and influence of using a semi-rigid seat over a real production seat, but rather as an indication of any limitations these requirements may pose in relation to submarining protection. While none of the three PMHSs in the 50 kph reclined experiments (UMTRI AVOK-Series, 2020) had a confirmed submarining outcome, they all had pelvic ring fractures with an incomplete/complete disruption of the posterior arch. Given the substantial pelvis damage, it is unclear how relevant classic submarining definitions are, and it is possible that the belt moved into the abdomen after breaking the pelvis without being considered submarining. Similarly, pelvic fractures from belt and seat loads have been recorded in other reclined PMHS tests utilizing a semi-rigid seat setup (Baudrit et al., 2022; Richardson et al., 2020; Shin et al., 2023). To design new occupant restraint systems that protects both against pelvic fracture and submarining, future evaluations might need a two-stage condition where a pass/fail check is independently applied to both fracture prediction and submarining outcome.

Expanding the evaluation from a single point pass/fail for submarining outcome, to a population-based risk evaluation, also opens the discussion for what can be considered “acceptable risk” when designing robust systems. This study does not aim to assess the post-submarining injury risk, since this is difficult to predict and can be dependent on factors not directly related to the submarining outcome, *e.g.*, head/knee impacts. Instead, risk is only considered for the submarining event itself, which should be used as the safety system design criteria. The threshold lines, separating submarining outcomes, developed in this study were generated based on the optimum metamodel classification accuracy identified through ROC at 0.37. This means that at the limit, a 37% probability of submarining is predicted with a TPR of 0.9 and a FPR of 0.07. From a safety standpoint it is more critical to identify the true positives (higher TPR), with a higher FPR as an acceptable tradeoff, which could be achieved by considering a lower threshold, albeit at the expense of model accuracy, in order to reduce the risk of missing an actual submarining outcome. Using a lower threshold would push the submarining threshold line to the right in Figure 10, *i.e.,* for a given seat pan angle, a greater buckle angle would be required to reach the estimated non-submarining zone. Furthermore, given that PA and ISH were both identified as significant parameters for submarining outcome, a critical pelvic shape for submarining could be defined based on population measurements to capture a pre-defined portion of the population, which could then be used as the submarining evaluation tool. More research, as well as ethical considerations, are required to define where a population boundary should be drawn and what could be considered an acceptable submarining probability for the critical occupant on this boundary.

### 4.3 Limitations

A semi-rigid seat was utilized in the present study in order to provide an easily parameterized environment, and to achieve a closer match with the reclined validation scenario. The semi-rigid seat was first presented by (Uriot et al., 2015) and is stated to replicate the behavior of a real front row seat used for frontal car crash evaluations, although no further details have been presented regarding validation or which specific seat model. Due to its ease of use and replication in FE simulations, the semi-rigid seat has been favored in multiple PMHS studies since its first introduction (Baudrit et al., 2022; Lopez-Valdes et al., 2024; Richardson et al., 2020; Shin et al., 2023; Somasundaram et al., 2022; 2023; UMTRI AVOK-Series, 2020), motivating a better understanding of expected PMHS response in this scenario. However, to the best of the authors’ knowledge, studies evaluating the semi-rigid seats capability of replicating the interaction between an occupant and a real production front row vehicle seat, has not been published to date, nor has how representative it is compared to a population of such seats. Evaluating hip kinematics in published frontal impact scenarios with upright PMHSs on production seats (Albert et al., 2018; Forman et al., 2006; Shaw et al., 2018), one can note that the hip tends to move down into the seat at peak forward excursion. For impact velocities around 50 kph and force limited belt systems, the downwards movement is approximately 30–50 mm. However, for the upright PMHS on a semi-rigid seat impacted at 50 kph (UMTRI AVOK-Series, 2020), the hip moves up by about 30–50 mm at peak forward excursion. Similar results were found when simulating the front seat configuration from (Uriot et al., 2015), but the PMHS vertical hip response was not published for comparison. In reclined PMHS experiments on a semi-rigid seat, the hip either moves up (UMTRI AVOK-Series, 2020) or is initially forced down by the lap belt pre-tensioner and then remains approximately horizontal until it interacts with the submarining ramp and is forced up (Baudrit et al., 2022; Richardson et al., 2020). These discrepancies between hip vertical motion in production seats *versus* the semi-rigid seat, indicate that the current implementation (current spring stiffness) of the semi-rigid seat produces a stiffer vertical response than real production seats, effectively forcing the hip to move towards the lap belt. This could result in excessive pelvic forces, increasing the risk of pelvic fracture, but also improved coupling between the pelvis and the lap belt, decreasing the risk of submarining. As such, this poses a limitation on the results of the present study regarding real-world relevance in an actual vehicle environment, which motivates further research on this topic. Since previous studies have already shown that small changes to the semi-rigid seat stiffness (±10% spring stiffness) had little effect on both pelvis kinematics and submarining outcome (Brynskog et al., 2024), and since it is currently unknown what spring stiffness would be representative of future seats designed and optimized for reclined occupants, it was decided to exclude seat stiffness as a parameter in this study.

This study is also limited by the fact that only the residual variance around the hip of a 50%ile male was considered. Previous studies have shown that a small female could be more sensitive to submarining outcomes (Rawska et al., 2019), and that obese occupants tend to have less favorable belt placement (Reed et al., 2012), which could also increase the risk of submarining. Hence, estimating the full effect of occupant *versus* restraint variability on submarining outcome is not possible, and the results presented herein most likely underpredict the influence of occupant variability had the complete population been considered. In addition, the full pelvic shape space was not considered since the analyzed pelvises were selected based on only two measurements, PA and HAD. This simplification was done since sampling from the full pelvic shape space presented in (Brynskog et al., 2021) would require 16 parameters only for the pelvis. Covering such a large design space when sampling becomes unfeasible given the limited number of simulations that could be performed. To partially overcome this limitation, a random pelvic shape fulfilling the select pelvis measurements was drawn for each evaluation point. Consequently, the 70 pelvises used for the metamodel simulations all represent conceivable 50%ile male pelvises, that fall within the 95% population boundary for both PA and HAD measurements. Furthermore, to avoid extreme shapes, the 16 parameters from (Brynskog et al., 2021) used to generate the random shapes were capped at ±2SD when drawing a random candidate. Similarly, due to the limitations in sampling the full design space, and lack of data describing complete population distributions for all parameters, some of the parameters were discretized. It is known that discretizing continuous variables with LHS can lead to a suboptimal design (Joseph et al., 2020), and that most likely, the sequential sample is not optimally space-filling, given the previous sample drawn. Furthermore, an efficient sampling strategy must be balanced between exploration (covering the complete design space) and exploitation (resolving the response in regions of interest) (Crombecq et al., 2011). The sequential method, of panning and zooming the parameter ranges to the region of greatest uncertainty, was chosen with the intention of targeting such a balance given the limited sample size.

The PMHSs of the 50 kph reclined validation (UMTRI AVOK-Series, 2020) all sustained both pelvis and ribcage fractures. While fracture mechanics can be included in FE-HBM simulations, fracture is a very chaotic and subject specific event that the SAFER HBM does not aim to capture, as it can be presupposed at this stage that the restraint system has already failed. Instead, the model targets predicting the risk of fracture using tools such as an IRF (Iraeus et al., 2023; Larsson et al., 2021). However, the kinematic response post-fracture can, therefore, be questioned and potentially influence the predicted submarining outcome. As summarized by (Richardson et al., 2024), pelvic fracture has been recorded in several PMHS studies at lap belt forces of approximately 5–8 kN. Seeing as the average anchor side lap belt force was 9.7 kN (SD = 0.9 kN) in this study, subjecting human beings to such loading scenarios would expose them to considerable risk of pelvic fractures.

Finally, the study only considered a pure frontal car crash at 50 kph, based on the pulse used in the PMHS tests (UMTRI AVOK-Series, 2020). Real-life crashes also include variability in severity, pulse shape, and impact angle, influencing occupant kinematics which could affect the submarining outcome.

## 5 Conclusion

Intrinsic occupant variance associated with a predicted 50%ile male can be comparable with the external restraint design variance’s effect on submarining outcome for reclined occupants. This indicates that future vehicle safety ratings using different versions of a 50%ile male FE-HBM, may be subject to variation in submarining outcome if harmonization of the target occupant anatomy/posture is not established. The parameter with the strongest effect was buckle angle, however, comparable effects were predicted for pelvis angle, H-Point forward/rearward placement, seat friction, and seat pan angle, while a weaker, but statistically significant, effect was found for the iliac spine hook angle.

The results indicate that to achieve robust submarining protection for reclined occupants, the current legal limits on buckle angle might need a shift towards more vertical angles. However, this result was achieved using a simplified seat model and additional studies are needed to evaluate if the identified effects hold in future production seats for reclined occupants. Furthermore, designing safe vehicles for reclined occupants will require consideration of both submarining risk as well as any risks associated with submarining prevention.

## Data Availability

The raw data supporting the conclusions of this article will be made available by the authors, without undue reservation.
