# Supplementary material for: Effect of occupant and restraint variability in reclined positions on submarining probability in frontal car crash scenarios
Source: Front Bioeng Biotechnol. 2025 Jul 9;13:1570572. doi: 10.3389/fbioe.2025.1570572 (PMC12283681; doi:10.3389/fbioe.2025.1570572)
Supplement: Supplementary file 1 [file DataSheet3.docx]

# Appendix C

Table 5 shows the position of the computed target (Reed & Ebert, 2018), positioned SAFER HBM, and gravity settled SAFER HBM, used as baseline in the sensitivity study. All measurements are defined as outlined in (Reed & Ebert, 2018), consequently the pelvis angle has not been defined as PA used for sampling, but rather as the angle between a vector from H-Point to center of S1L5 with respect to vertical.

Table 5 – Baseline FE-HBM position for the sensitivity study. Computed target based on regression equations in (Reed & Ebert, 2018), for a subject with stature = 1.75 m, BMI = 25 kg/m2, and sitting height to stature (SHS) ratio = 0.52, on a seat with 45° back angle including a head rest, positioned SAFER HBM pre gravity load, gravity settled SAFER HBM, and difference between target and gravity settled SAFER HBM..
*Measurements defined as outlined in (Reed & Ebert, 2018).

| **Measurement*** | **Computed target** | **Positioned** | **Gravity Settled** | **Target to Gravity Settled Diff.** |
| --- | --- | --- | --- | --- |
| Pelvis angle | 65.4° | 65.4° | 78.1° | +12.7° |
| Lumbar angle | 38.6° | 39.1° | 40.3° | +1.7° |
| Thorax angle | 33.4° | 33.5° | 31.2° | -2.2° |
| Neck angle | 32.1° | 31.7° | 32.2° | +0.1° |
| Head angle | 23.8° | 23.4° | 23.9° | +0.1° |
| Thigh angle | 11.7° | 11.2° | 11.5° | +0.2° |
| Leg angle | 49.1° | 48.7° | 47.1° | -2.0° |
| Hip-eye angle | 31.1° | 30.3° | 30.9° | -0.2° |
| Hip-eye X | 336 mm | 343 mm | 340 mm | +4 mm |
| Hip-eye Z | 567 mm | 587 mm | 568 mm | +1 mm |

## References

Reed, M. P., & Ebert, S. M. (2018). Effects of Recline on Passenger Posture and Belt Fit. *University of Michigan Transportation Research Institute, Report No. UMTRI-2018-2*. <https://deepblue.lib.umich.edu/bitstream/handle/2027.42/146263/UMTRI-2018-2.pdf?sequence=1&isAllowed=y>
