# Supplementary material for: Effect of occupant and restraint variability in reclined positions on submarining probability in frontal car crash scenarios
Source: Front Bioeng Biotechnol. 2025 Jul 9;13:1570572. doi: 10.3389/fbioe.2025.1570572 (PMC12283681; doi:10.3389/fbioe.2025.1570572)
Supplement: Supplementary file 2 [file DataSheet2.docx]

# Appendix B

**Additional information regarding the parameter space**

**Pelvic shape sampling**

Figure *20* shows the pelvic shape sample with black contours representing the baseline and random 50%ile male pelvises at the 95%ile population boundary for PA and HAD, used in Batch 1 of the sensitivity study. Each additional evaluation point (n = 69) inside the population boundary (green and yellow circles) is associated with additional random pelvic shapes matching the corresponding PA and HAD measurements. All pelvises were aligned to a common sacral endplate position and angle since the spine was kept with a constant curvature.

| 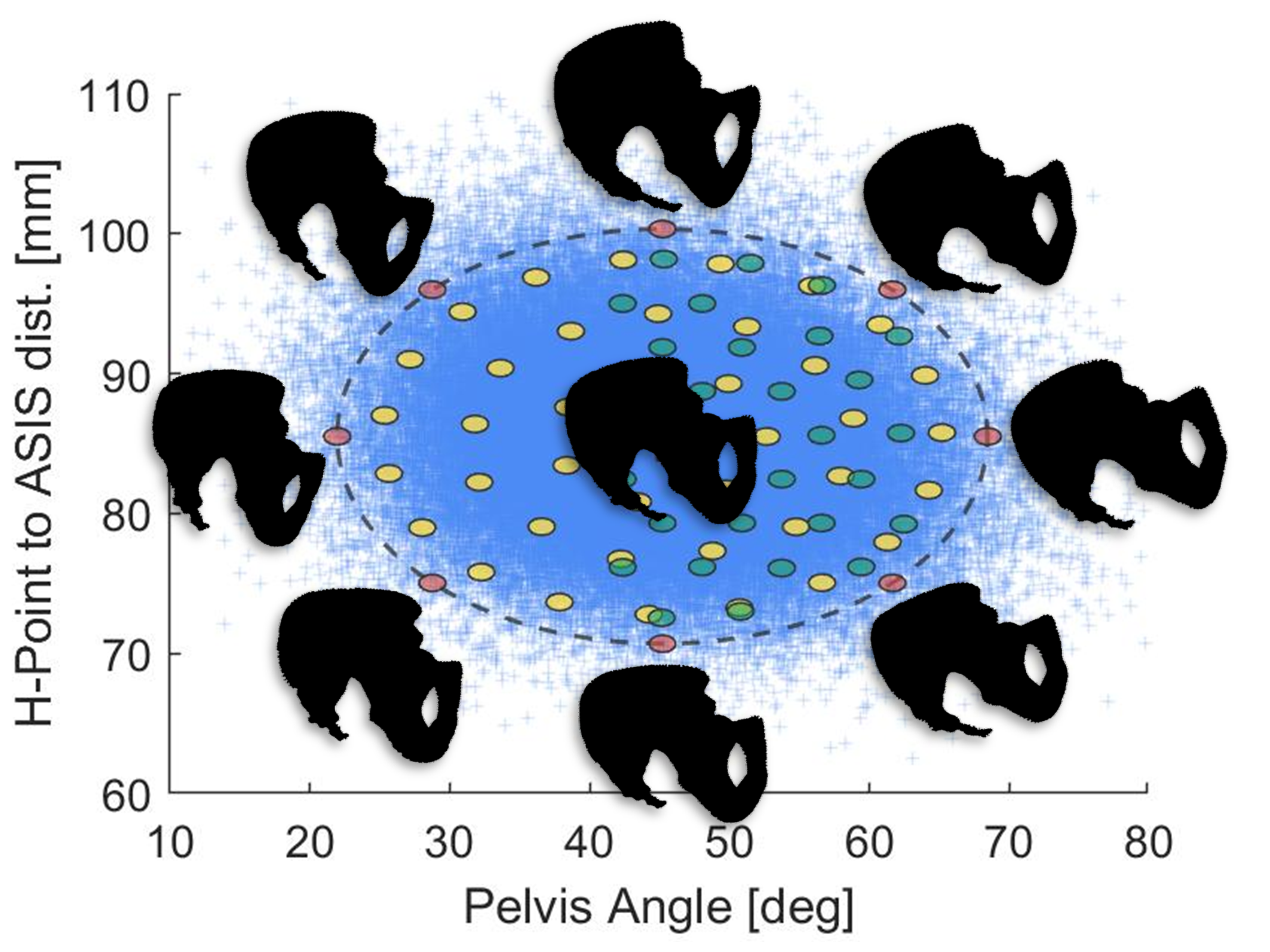 |
| --- |

Figure 20 – Pelvic shape sample with black contours of the baseline pelvis (middle) and on the 95%ile population boundary used for Batch 1 simulations (red circles).

**Sampled PA versus resulting PA**

As mentioned in the Results, the resulting PA (measured after gravity settling in a reclined position) was chosen over the sampled PA (sampled in the baseline SAFER HBM position) as a predictor for the metamodel. A correlation score between these measurements of 0.94 was found from the sensitivity study simulations. Figure *21* shows the correlation between sampled PA and resulting PA.

| 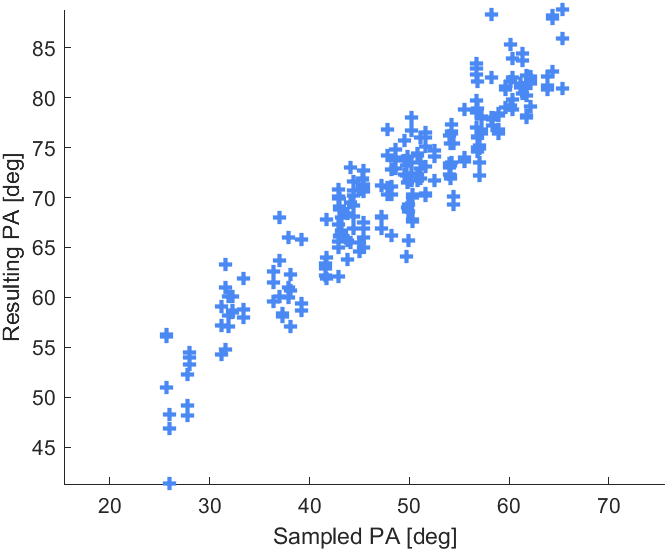 |
| --- |

Figure 21 – Comparison of sampled PA versus resulting PA after gravity settling on seat in reclined position (one cross for each simulation, n = 240).

**HAD versus ISH**

As mentioned in the Results section, the ISH was chosen over HAD as predictor for the metamodel, motivated by the unintuitive effect identified where greater HAD (essentially more pelvis surface for the belt to catch) resulted in higher probability for submarining. A correlation score between HAD and ISH of 0.71 was found through the sensitivity study simulations. Hence, a greater HAD was associated with a greater ISH, *i.e.*, a flatter iliac spine. This means that even though the belt had more surface to catch, it was not enough to compensate for the fact that there was also less of a hook at the ASIS where the belt could stick. Figure *22* shows the correlation between HAD and ISH.

| 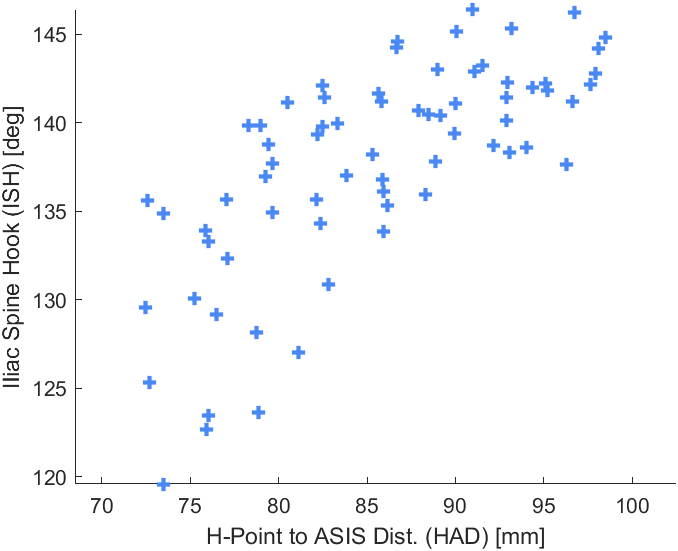 |
| --- |

Figure 22 – Comparison of HAD versus ISH from sensitivity study models (one cross for each pelvic shape, n = 70).

**Soft tissue thickness**

As mentioned in the Methods and Material section, the soft tissue thickness around the pelvis was defined as three discrete levels (-2RMSE / baseline / +2RMSE) based on a linear regression model of circumference of male buttocks from the ANSUR 2 data (Gordon et al., 2014). The linear regression model was generated on the male dataset using the natural logarithm of *“buttockcircumference”* as continuous response and normalized (mean = 0, SD = 1) *“staturemm”* and *“BMI”* as predictors. Table 4 shows the resulting regression model with normalized stature and BMI. The model has an adjusted R^2^ = 0.88.

Table 4 – Linear regression model of log(buttock circumference) with normalized (mean = 0, SD = 1) predictors.

|  | **Estimate** | **SE** | **p-val.** |
| --- | --- | --- | --- |
| *Intercept* | 6.9243 | 0.0004 | <0.001 |
| Stature_Normalized_ | 0.0254 | 0.0004 | <0.001 |
| BMI_Normalized_ | 0.0657 | 0.0004 | <0.001 |

Using the regression model with the 50%ile SAFER HBM anthropometry (stature = 1750 mm, BMI = 25 kg/m^2^), circumference of average buttocks was predicted as 971 mm (95% CI: 922 – 1022 mm). To cover 95% of the population (±2RMSE), this resulted in a -5.0/+5.3% scaling on the predicted circumference. Simplifying the buttock circumference as a circle, this scaling equals a radius change of -7.7/+8.2 mm, which was achieved by normal projection of the elements surrounding the hip. Figure *23* shows the resulting scaled models together with the baseline.

| 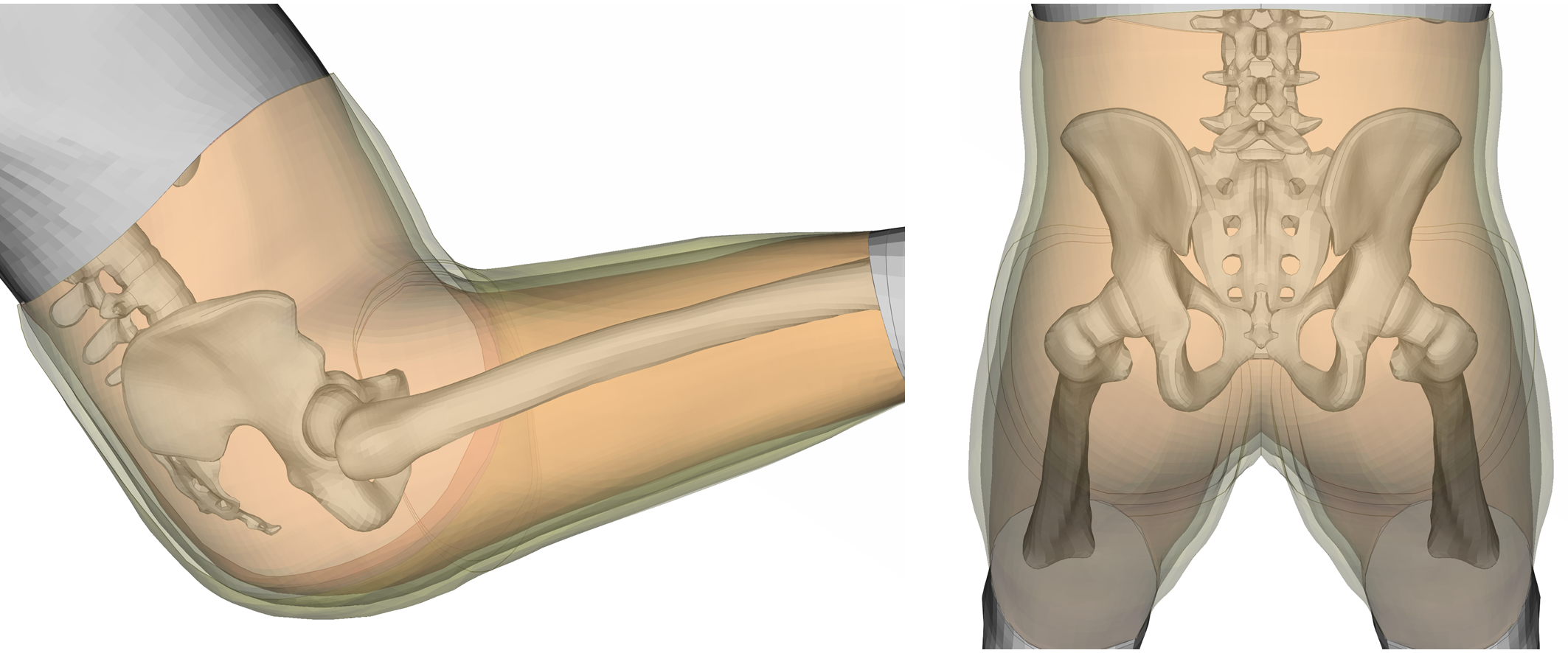 |
| --- |

Figure 23 – Baseline (gray) and ±2RMSE circumference of buttocks to have three versions of soft tissue thickness around the hip and thigh.

## References

Gordon, C. C., Blackwell, C. L., Bradtmiller, B., Parham, J. L., Barrientos, P., Paquette, S. P., Corner, B. D., Carson, J. M., Venezia, J. C., Rockwell, B. M., Mucher, M., & Kristensen, S. (2014). *2012 Anthropometric Survey of U.S. Army Personnel: Methods and Summary Statistics. Technical Report NATICK/TR-15/007*.
