# Supplementary material for: Effect of occupant and restraint variability in reclined positions on submarining probability in frontal car crash scenarios
Source: Front Bioeng Biotechnol. 2025 Jul 9;13:1570572. doi: 10.3389/fbioe.2025.1570572 (PMC12283681; doi:10.3389/fbioe.2025.1570572)
Supplement: Supplementary file 3 [file DataSheet1.docx]

# Appendix A

**Validation Method**

A simulation environment was created including sled, distant foot and knee support, semi-rigid seat (Uriot et al., 2015), and a 3-point belt with buckle, latch, and shoulder belt retractor with pre-tensioner and load limiter functionality. The belt was modeled with eight elements across the width, using quadrilateral under-integrated membrane (ELFORM = 5) elements with a thickness of 1.2 mm and a 2D seatbelt material (*MAT_SEATBELT_2D) with default settings, similar to (Brynskog et al., 2024). The loading/unloading curves for the belt material were based on data from a generic FE vehicle buck model (Iraeus & Lindquist, 2016), scaled to produce 10.1 kN belt force at 10% stretch. The belt filmspool effect, pre-tensioner force and timing (1.4/1.7 kN, triggered 10 ms after acceleration onset with an 8 ms linear ramp), and load limiter force (3.4/2.7 kN at 32/50 kph, respectively) were modeled to match the reported belt force of the experiments.

To achieve a positioned model, the tracked data from the experiments at time zero for the head, T1, T8, T12, shoulder, hip, ankle, and belt system, was compiled and averaged over all subjects in the upright/reclined (25°/45°) scenarios, respectively. Together with the reported target positioning equations (Reed et al., 2019), the SAFER HBM was positioned using the “Human Body Model – Articulation” tool in ANSA. The positioned models were then de-penetrated in all contacts and checked for negative volume elements.

To initiate the simulations, the model was aligned in X-direction with the seat H-point and moved up in Z-directions until there was no contact between the FE-HBM and the seat pan. A gravity load with global damping and constraints on the FE-HBM in the sagittal plane, including pelvis rotation for the validation setup, was applied to reach equilibrium in 300 ms of simulation time, before ramping down the damping and applying the actual load (32/50 kph pulse). See Table 3 for a comparison of average PMHS and gravity settled FE-HBM positions, and Figure *11* for the gravity settled upright / reclined models and the response at approximate peak hip displacement for the 50 kph pulse.

Table 3 – Average PMHS position from upright/reclined scenario compared to gravity settled SAFER HBM position.

|  | **Upright (25° back angle)** | | **Reclined (45° back angle)** | |
| --- | --- | --- | --- | --- |
| **Measurement** | **Average PMHS** | **SAFER HBM** | **Average PMHS** | **SAFER HBM** |
| Pelvis angle | 62.6° | 62.8° | 68.9° | 69.3° |
| Thorax angle | 14.3° | 12.8° | 37.8° | 37.1° |
| Head angle | 6.4° | 5.3° | 23.2° | 22.9° |
| H-Point X rel. seat H-Point | 4 mm | 5.9 mm | 0 mm | 6.3 mm |
| T12 Spinous Process X | -251 mm | -254 mm | -276 mm | -270 mm |
| T1 Spinous Process X | -325 mm | -318 mm | -461 mm | -443 mm |
| Infraorbital X | -159 mm | -157 mm | -345 mm | -337 mm |

| 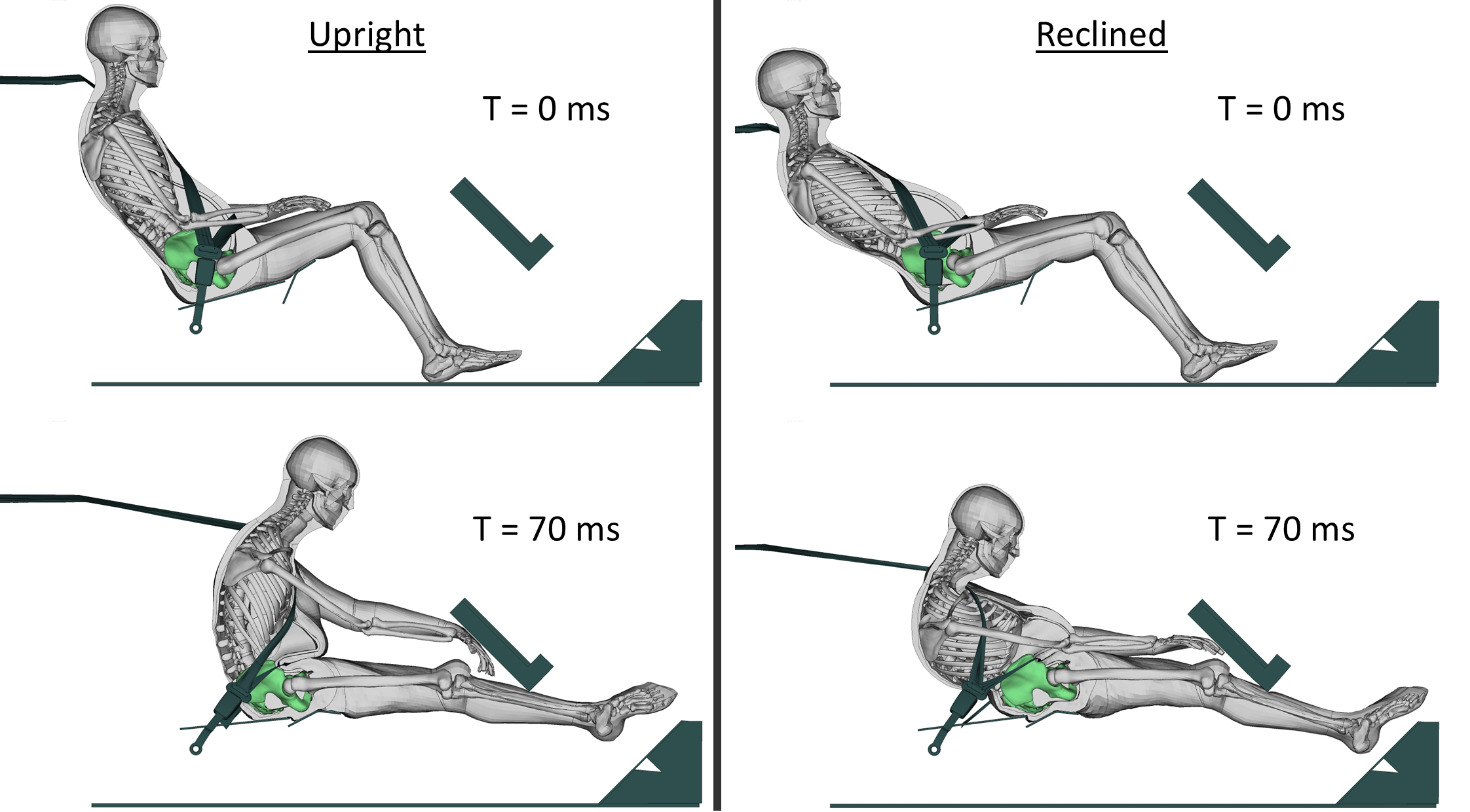 |
| --- |

Figure 11 – Upright (left) and reclined (right) gravity settled models (top row) and at approximately peak forward hip excursion (bottom).

**Validation Results**

Figure *12* to Figure *19* shows the boundary conditions and kinematic signals of the baseline/scaled 50%ile SAFER HBM in an upright/reclined posture at 32/50 kph impact speed compared to the PMHS response. The simulation response was qualitatively compared to the reported experiment data, since missing data in the tracked signals did not allow for a quantitative evaluation such as the CORrelation and Analysis method (CORA) (Gehre et al., 2009).

Overall, the SAFER HBM predicted the PMHS response with main kinematic deviations identified as hyper flexion of the head and lower spine Z-displacement in the 32 kph reclined scenario. By studying the video of the PMHS experiments it was noted that the PMHSs seemed to interact with the low back support used to position the pelvis as it moved forward in the 32 kph reclined scenario, this was not observed in any of the other scenarios. It is hypothesized that this interaction caused the PMHS to keep a higher T12 vertical position (less positive Z-displacement) than the simulated response. Due to the uncertainty of this interaction, and lack of reported data regarding the back support, this feature was not included in the simulation model.

The main deviations for boundary conditions were peak lap belt force in 50 kph scenarios, peak seat rotation and rebound, and buckle rebound in 50 kph scenarios. It is hypothesized that the peak lap belt force was somewhat overpredicted in the 50 kph scenarios due to the lack of simulated pelvic fractures, which could have an unloading effect on the lap belt. The seat stiffness was validated against reported data from UMTRI, however, the seat rotation did not fully match the experiments despite a good match in seat force, and the reason for this discrepancy remains unclear.

| 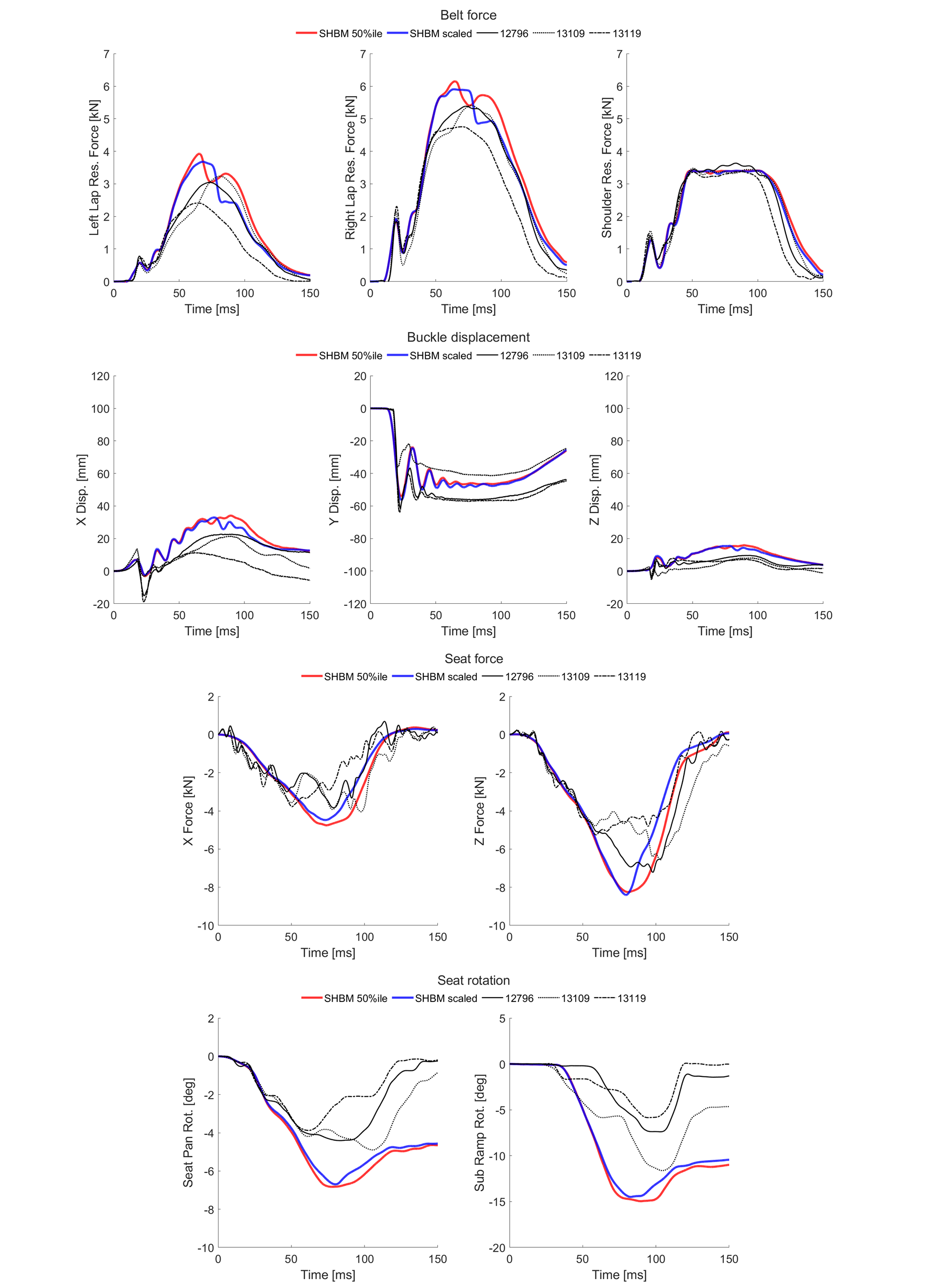 |
| --- |

Figure 12 – Boundary condition signals for the baseline (red) and scaled (blue) 50%ile SAFER HBM compared to PMHS response (black) in the upright 32 kph scenario.

| 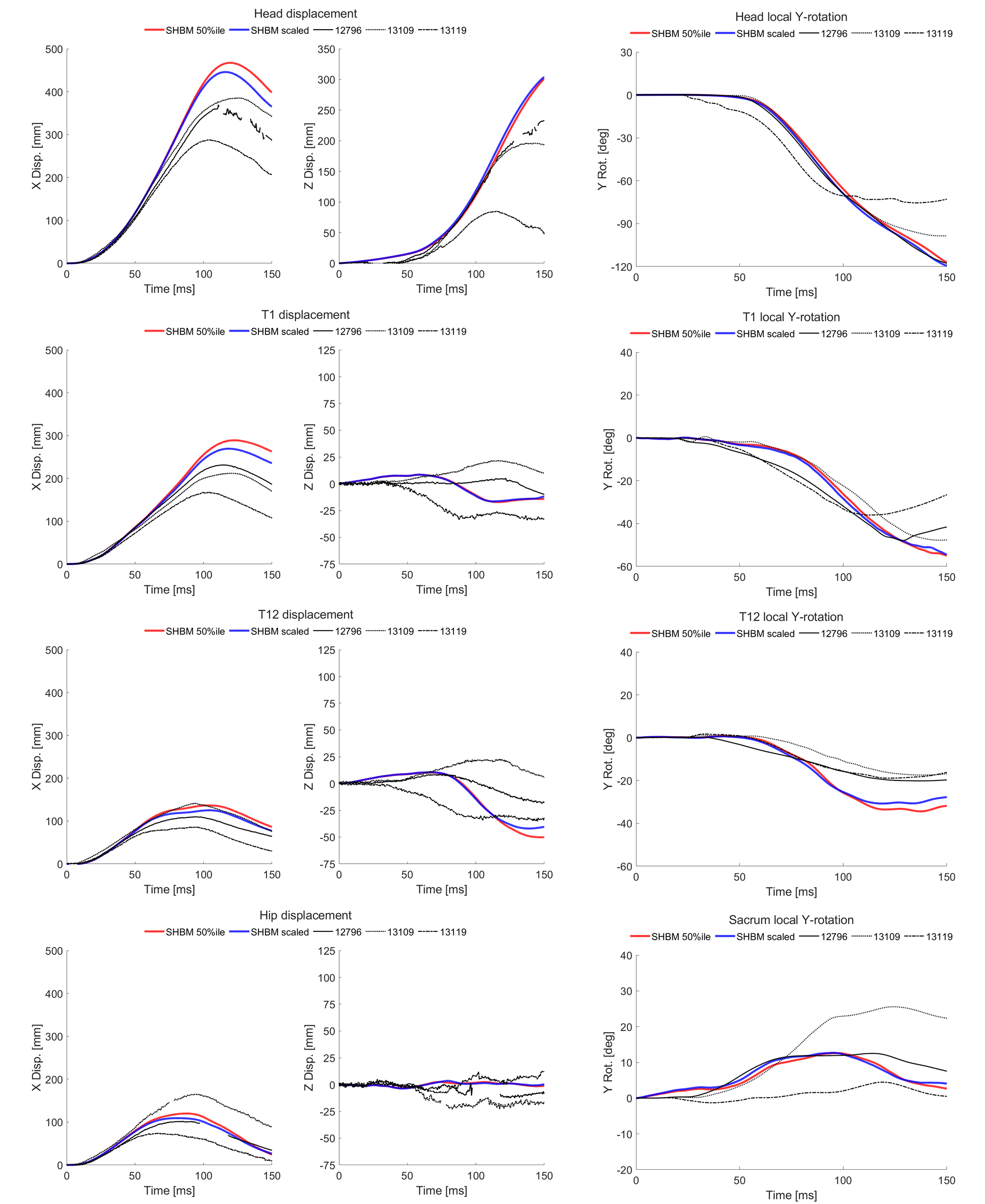 |
| --- |

Figure 13 – Kinematic signals for the baseline (red) and scaled (blue) 50%ile SAFER HBM compared to PMHS response (black) in the upright 32 kph scenario.

| 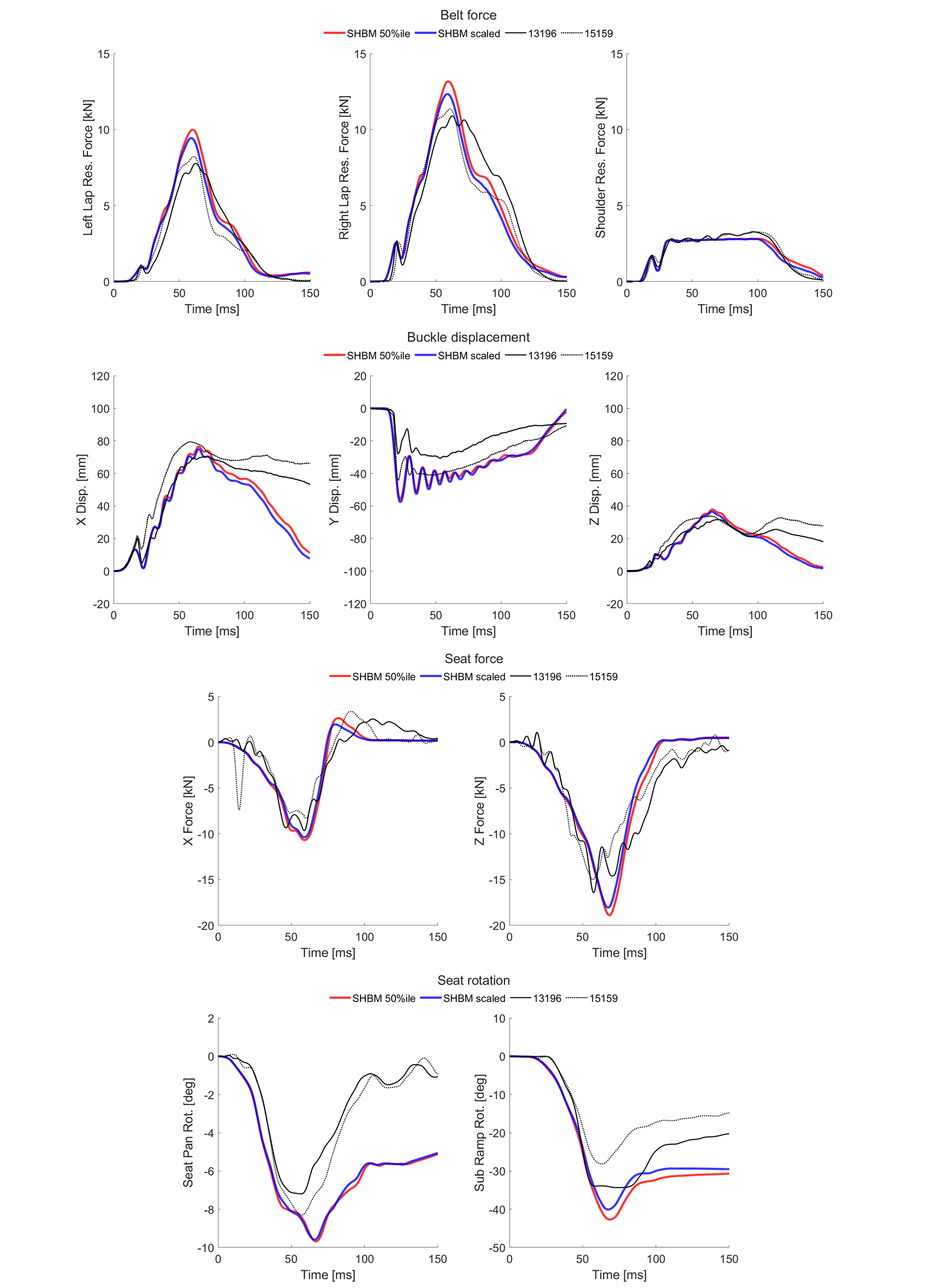 |
| --- |

Figure 14 – Boundary condition signals for the baseline (red) and scaled (blue) 50%ile SAFER HBM compared to PMHS response (black) in the upright 50 kph scenario.

| 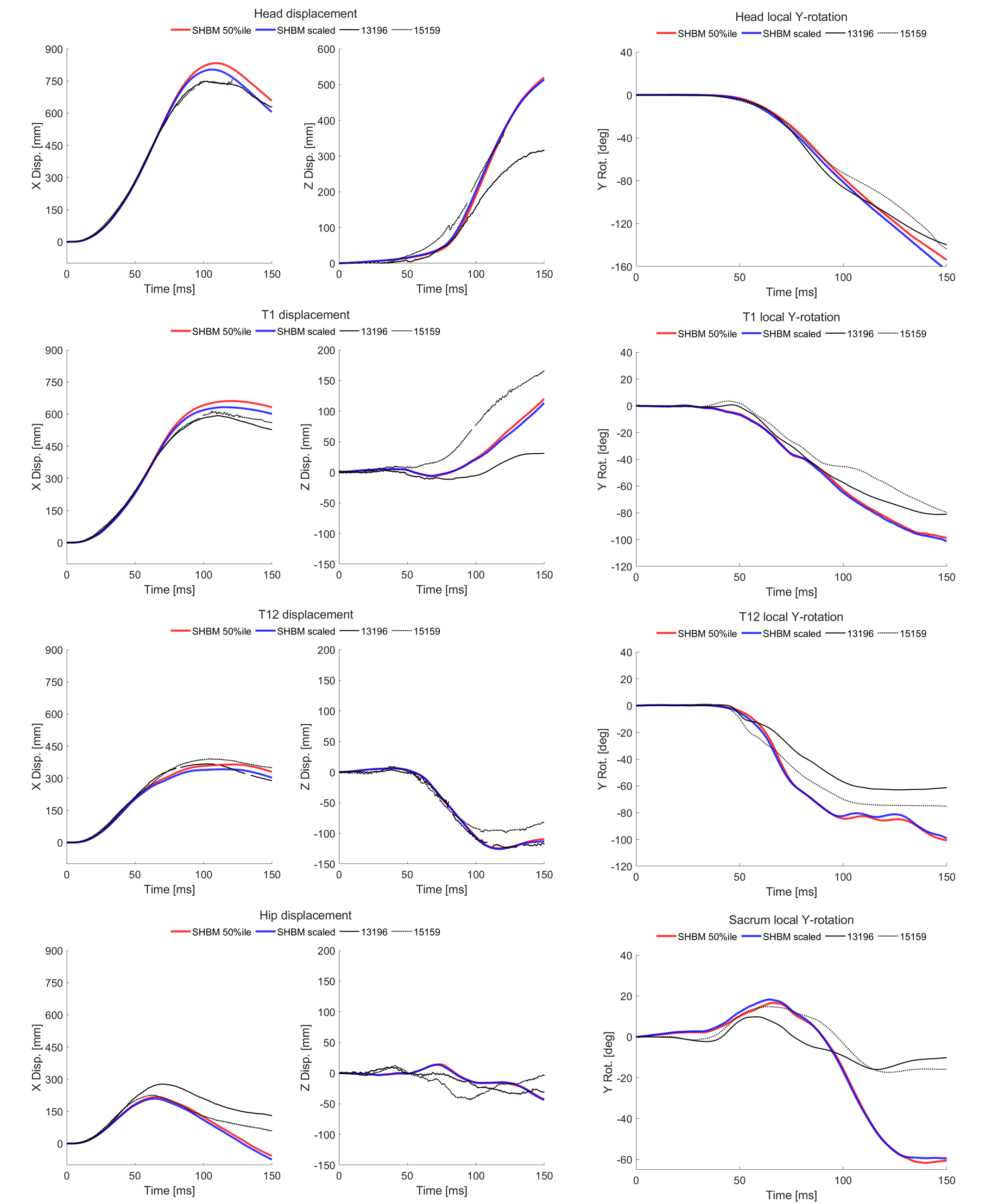 |
| --- |

Figure 15 – Kinematic signals for the baseline (red) and scaled (blue) 50%ile SAFER HBM compared to PMHS response (black) in the upright 50 kph scenario.

| 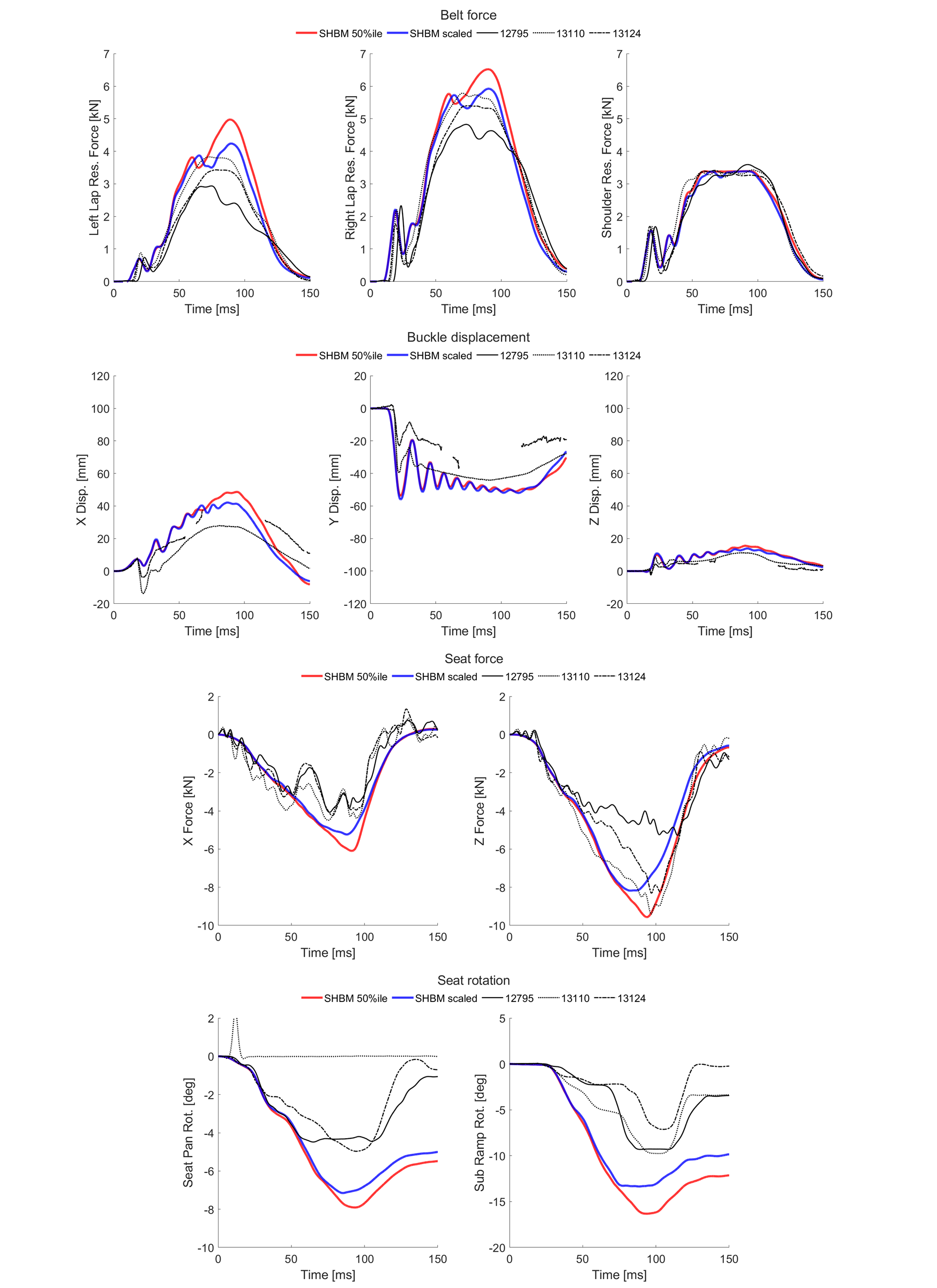 |
| --- |

Figure 16 – Boundary condition signals for the baseline (red) and scaled (blue) 50%ile SAFER HBM compared to PMHS response (black) in the reclined 32 kph scenario.

| 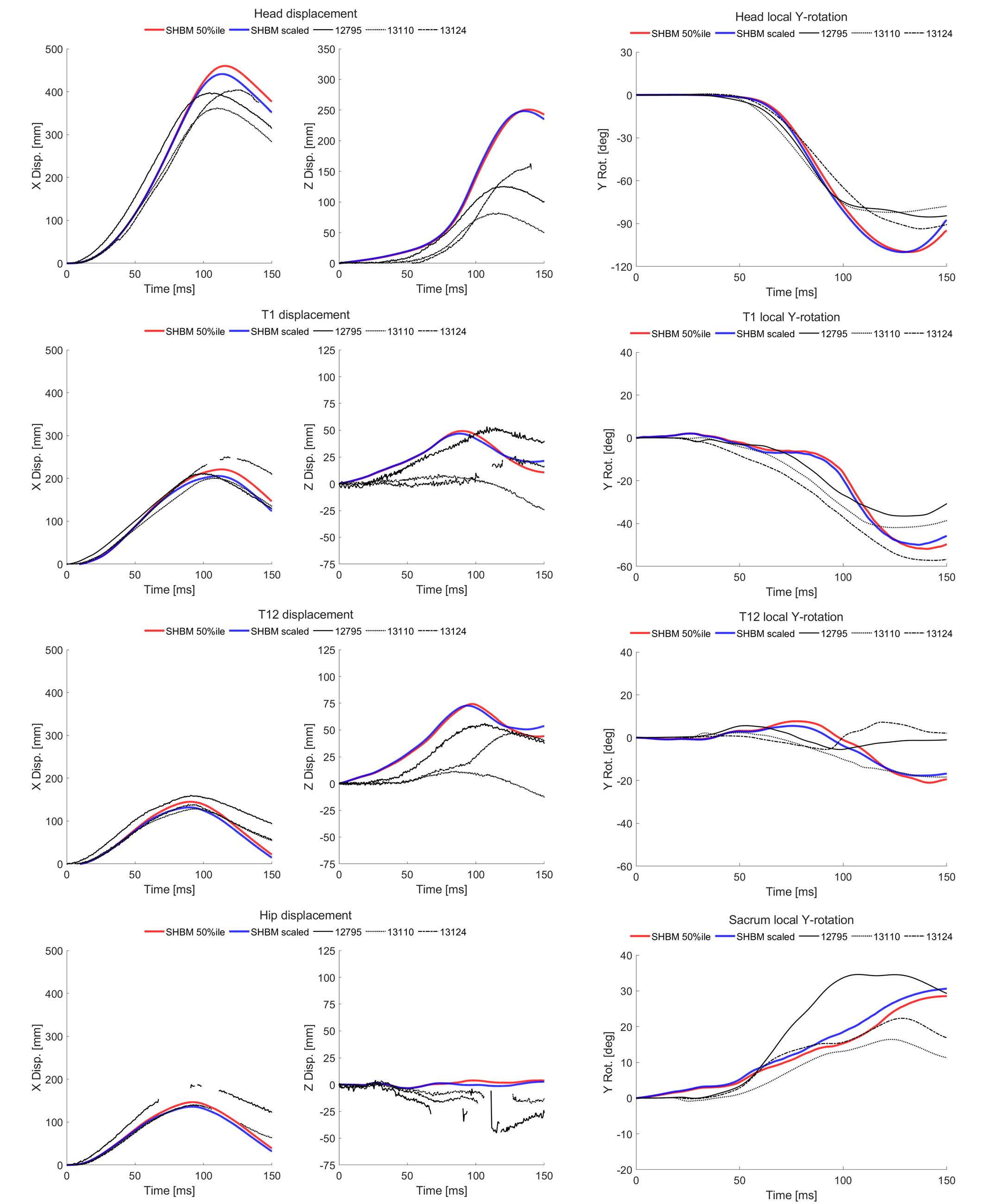 |
| --- |

Figure 17 – Kinematic signals for the baseline (red) and scaled (blue) 50%ile SAFER HBM compared to PMHS response (black) in the reclined 32 kph scenario.

| 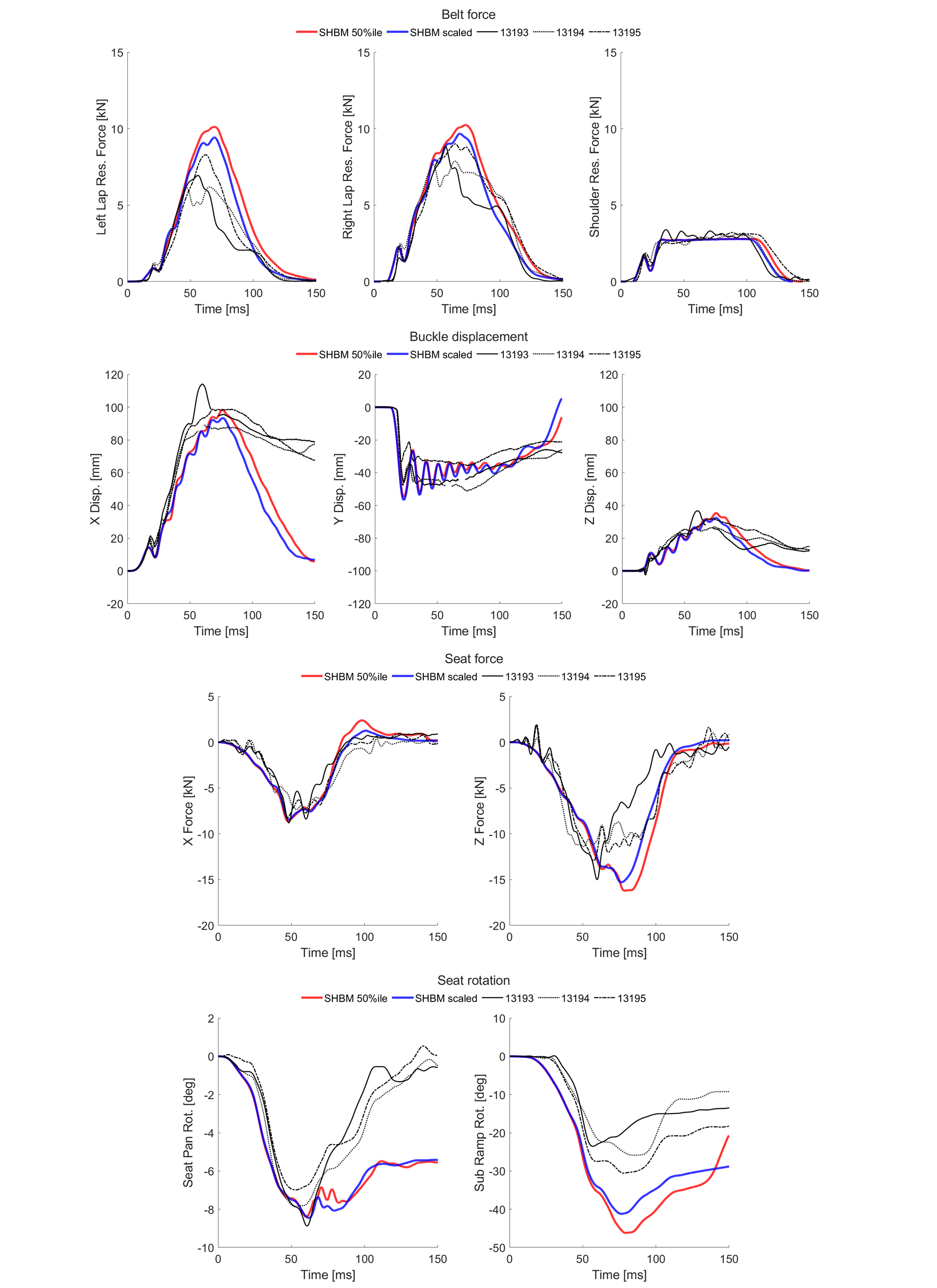 |
| --- |

Figure 18 – Boundary condition signals for the baseline (red) and scaled (blue) 50%ile SAFER HBM compared to PMHS response (black) in the reclined 50 kph scenario.

| 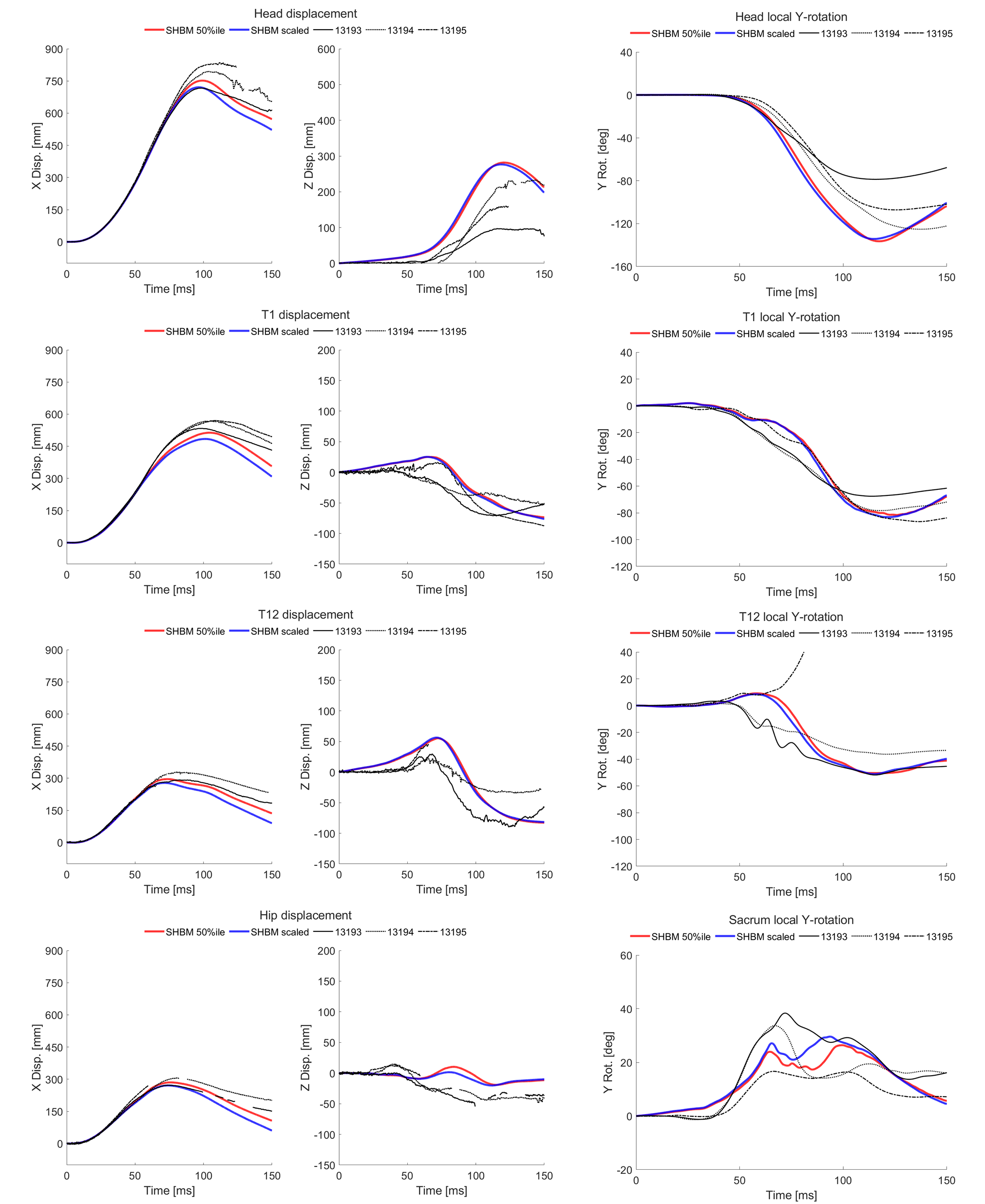 |
| --- |

Figure 19 – Kinematic signals for the baseline (red) and scaled (blue) 50%ile SAFER HBM compared to PMHS response (black) in the reclined 50 kph scenario.

## References

Brynskog, E., Iraeus, J., Pipkorn, B., & Davidsson, J. (2024). Simulating Pelvis Kinematics from Belt and Seat Loading in Frontal Car Crash Scenarios: Important Boundary Conditions that Influence the Outcome. *Annals of Biomedical Engineering*, 1–30. <https://doi.org/10.1007/S10439-024-03631-9/FIGURES/19>

Gehre, C., Gades, H., & Wernicke, P. (2009). ”Objective Rating of Signals Using Test and Simulation Responses,” in *ESV Conference*.

Iraeus, J., & Lindquist, M. (2016). Development and validation of a generic finite element vehicle buck model for the analysis of driver rib fractures in real life nearside oblique frontal crashes. Accident Analysis and Prevention, 95, 42–56. <https://doi.org/10.1016/j.aap.2016.06.020>

Reed, M. P., Ebert, S. M., & Jones, M. L. H. (2019). Posture and belt fit in reclined passenger seats. Traffic Injury Prevention, 20(sup1), S38–S42. <https://doi.org/10.1080/15389588.2019.1630733/SUPPL_FILE/GCPI_A_1630733_SM3037.PDF>

Uriot, J., Potier, P., Baudrit, P., Trosseille, X., Petit, P., Richard, O., Compigne, S., Masuda, M., & Douard, R. (2015). Reference PMHS Sled Tests to Assess Submarining. Stapp Car Crash Journal, 59, 203–223.
